# Effect of AlCrFeCuNi High-Entropy Alloy Reinforcement with a Nanocrystalline Internal Structure on the Microstructure, Electrical Conductivity and Tribological Behavior of SPS-Processed Cu–B_4_C Composites

**DOI:** 10.3390/nano16181190

**Published:** 2026-09-21

**Authors:** Müslim Çelebi

**Affiliations:** Department of Metallurgical and Materials Engineering, Faculty of Engineering, Karadeniz Technical University, 61080 Trabzon, Türkiye; muslimcelebi@ktu.edu.tr

**Keywords:** nanocrystalline high-entropy alloy, AlCrFeCuNi, Cu matrix composite, tribology, SPS

## Abstract

This study investigates the effect of mechanically alloyed AlCrFeCuNi high-entropy alloy (HEA) reinforcement with a nanocrystalline internal structure on the microstructural, mechanical, electrical, and tribological properties of Cu–1 wt.% B_4_C composites containing 0–30 wt.% HEA. The AlCrFeCuNi reinforcement used in this work had previously been produced by 25 h of mechanical alloying and characterized in detail, exhibiting a dual FCC–BCC structure and an average crystallite size of 10.2 nm while retaining micrometer-scale particle dimensions. The Cu–B_4_C–HEA powder mixtures were subsequently milled and consolidated by spark plasma sintering at 800 °C under 35 MPa. Increasing the HEA content progressively refined the powder mixture and increased the hardness from 78.8 HB for 0HEA to 138.94 HB for 20HEA, while the latter retained a relative density of 96.1% and an electrical conductivity of 67% IACS. The 20HEA composite exhibited the lowest average friction coefficient (0.36) and specific wear rate (1.01 × 10^−3^ mm^3^/N·m), corresponding to an approximately 88% reduction in wear rate relative to 0HEA. Microstructural and worn-surface analyses showed that this behavior was associated with the combined effects of hard HEA reinforcement, preserved Cu-matrix continuity, and the formation of an oxygen-rich mechanically mixed layer. Increasing the HEA content to 30 wt.% promoted reinforcement clustering and residual porosity, which reduced densification and partially deteriorated the tribological performance.

## 1. Introduction

Particle-reinforced metal matrix composites (MMCs) have found extensive use in aerospace, automotive, energy, and electronic applications because they can combine high functional performance with relatively favorable production costs [1,2]. Their ability to maintain stable properties while allowing mechanical and physical characteristics to be tailored through compositional and microstructural design has also driven substantial research interest [3,4]. Within this class of materials, particle-reinforced copper matrix composites are particularly attractive for applications requiring a combination of mechanical reliability and electrical or thermal conductivity [5,6]. Conventional copper alloys, however, are constrained by limited strength at elevated temperatures and by the difficulty of simultaneously improving mechanical properties without sacrificing conductivity [7,8]. In copper matrix composites, the resulting performance is governed mainly by the characteristics of the copper matrix, the type and distribution of the reinforcing particles, and the quality of the matrix–reinforcement interface. Appropriate control of these factors provides a practical route to overcoming several inherent limitations of conventional copper alloys [9,10]. The choice of reinforcement phase is a key factor in the design of particle-reinforced copper matrix composites. Common reinforcements include ceramic particles, metallic glasses, intermetallic compounds, and amorphous alloys [11,12]. Among ceramic reinforcements, B_4_C is particularly attractive because of its high hardness, low density, and resistance to abrasive wear. However, increasing the B_4_C content may introduce processing difficulties arising from its poor deformability, limited wettability with Cu, reduced densification, particle agglomeration, and disruption of the continuous Cu matrix [13,14]. These effects can restrict the simultaneous improvement of mechanical and functional properties, especially when electrical conductivity must also be preserved. Therefore, the B_4_C content in the present study was fixed at a low level of 1 wt.% rather than increased further, allowing it to provide a constant hard ceramic contribution while limiting the adverse effects associated with higher ceramic fractions.

High-entropy alloys (HEAs), generally composed of several principal elements in near-equiatomic proportions, can form relatively simple solid-solution structures [15,16]. Their compositional and structural characteristics can provide high strength, hardness, ductility, thermal stability, and corrosion resistance [17,18]. Unlike ceramic reinforcements, their metallic nature can also promote compatibility with Cu and reduce the large interfacial and thermal-expansion mismatch commonly encountered in ceramic-reinforced composites [11,14]. Several studies have demonstrated the effectiveness of this approach. Zhu et al. [19] fabricated FeCoNiCrAl HEA particle-reinforced Cu matrix composites by friction stir processing and reported a 69.8% increase in microhardness and a 29.7% reduction in wear rate, while the composite retained approximately 87% of the thermal conductivity of unreinforced Cu. Zhang et al. [1] prepared FeCoCrNi HEA-reinforced Cu matrix composites by ball milling followed by spark plasma sintering and showed that HEA addition simultaneously affected mechanical and transport properties. The 20 wt.% HEA/Cu composite reached a yield strength of 164 MPa, while the 15 wt.% composite exhibited a tensile strength of 283 MPa.

AlCrFeCuNi is a Co-free multicomponent high-entropy alloy characterized by a multiphase constitution in which FCC- and BCC-type solid solutions can coexist [20]. This dual-phase structure provides a relatively hard and mechanically stable metallic reinforcement while preserving the deformability and interfacial compatibility associated with metallic phases [21]. Experimental studies on mechanically alloyed and SPS-consolidated AlCrCuFeNi have confirmed the coexistence of FCC and BCC phases together with high hardness and strength, supporting its suitability for mechanically demanding composite systems [22]. The AlCrFeCuNi powder employed in the present study had previously been produced by 25 h of mechanical alloying and characterized in detail in our previous work [21]. That study established a dual FCC–BCC phase constitution, an average crystallite size of 10.2 nm, and the corresponding lattice parameters and hardness of the mechanically alloyed HEA. Although the powder particles remained in the micrometer-size range, the crystallite-size analysis confirmed a nanocrystalline internal structure. This distinction between particle size and crystallite size is particularly relevant when mechanically alloyed HEAs are employed as metallic reinforcements in bulk composites. The presence of Cu within the alloy is particularly relevant for Cu-based composites because a common constituent in the reinforcement and matrix can facilitate mutual diffusion and improve interfacial bonding compared with conventional ceramic reinforcements [23]. This concept has been demonstrated in related AlCrCuFeNi-based composite systems. Chen et al. [24] incorporated AlCrCuFeNi_2.5_ HEA particles into a Cu matrix by ball milling and spark plasma sintering; the Cu–20 wt.% HEA composite reached a compressive strength of 920 MPa while retaining an electrical conductivity of 44.3% IACS. Strong interfacial bonding between the HEA particles and Cu was identified as a major factor governing the mechanical–electrical property balance. Özkaya et al. [22] reinforced ZA40 alloy with 5–20 wt.% AlCrCuFeNi HEA particles and reported that increasing the HEA content to 20 wt.% increased hardness from 132 to 205 HB and tensile strength from 156 to 255 MPa. Under a 20 N load, the same reinforcement level produced an approximately 94.7% reduction in wear loss compared with the unreinforced ZA40 alloy, demonstrating a substantial improvement in wear resistance.

Spark plasma sintering (SPS) is an advanced powder metallurgy technique that has been extensively employed for the consolidation of particle-reinforced metal matrix composites [23,25]. The process simultaneously applies uniaxial pressure and pulsed electric current, generating Joule heating within the powder compact and promoting rapid atomic diffusion and interparticle bonding [26]. A distinctive feature of SPS is the localized Joule heating generated at particle–particle contact regions, where concentrated electrical resistance produces highly localized thermal fields that accelerate neck nucleation and growth [27]. This contact-scale thermal activation, coupled with externally applied pressure, markedly enhances densification kinetics and permits consolidation at lower bulk temperatures and considerably shorter holding times than conventional pressureless sintering. The abbreviated thermal exposure inherent to SPS restricts excessive grain coarsening and favors the retention of refined microstructural features, while accelerated diffusion and neck development promote pore elimination and structural homogenization. Localized electrical effects at interparticle interfaces may further assist in disrupting surface oxide films and increasing the effective contact area between adjacent particles, thereby improving interfacial continuity during consolidation. Such processing conditions are particularly relevant for Cu-matrix composites containing mechanically alloyed HEA reinforcement because effective consolidation must be achieved while limiting excessive microstructural coarsening, reinforcement agglomeration, and detrimental interfacial reactions [25,26,28].

Previous studies have established the feasibility of using HEA particles as reinforcements in Cu matrices, but the closest reported systems address different reinforcement architectures and property relationships. Zhu et al. [19] investigated FeCoNiCrAl-reinforced Cu surface composites produced by friction stir processing, whereas Zhang et al. [1] examined FeCoCrNi/Cu composites produced by ball milling and SPS with emphasis on mechanical, electrical, thermal, and thermoelectric properties. Chen et al. [24] investigated SPS-processed Cu/AlCrCuFeNi_2.5_ composites primarily from the perspective of interfacial strengthening and the strength–conductivity balance. More closely related to the present material, Yanar et al. [23] studied Cu–AlCrFeCuNi–graphene composites processed by SPS; however, the AlCrFeCuNi content was maintained at 20 wt.% while the graphene fraction was varied. Thus, the influence of the AlCrFeCuNi fraction itself on the coupled evolution of densification, Cu-matrix continuity, mechanical response, electrical conductivity, and tribological behavior in a ceramic-containing hybrid Cu composite has not been isolated by these studies.

The experimental design adopted here addresses this issue by maintaining B_4_C at a constant low level of 1 wt.% while systematically varying the mechanically alloyed AlCrFeCuNi HEA content from 0 to 30 wt.%. Fixing the B_4_C fraction provides a constant ceramic contribution and allows the composition-dependent role of the metallic HEA reinforcement to be distinguished from changes associated with ceramic content. The principal contribution of this study is therefore to establish the transition from beneficial HEA reinforcement to reinforcement-induced microstructural deterioration: up to 20 wt.% HEA, increasing reinforcement content improves hardness and wear resistance while preserving relatively high densification and Cu-matrix continuity, whereas at 30 wt.% HEA, increased particle clustering, HEA–HEA contacts, residual porosity, and loss of Cu-matrix continuity offset the strengthening benefit. By correlating these microstructural changes with hardness, electrical conductivity, friction, and wear, the present study identifies the composition-dependent balance governing the multifunctional performance of Cu–1 wt.% B_4_C–AlCrFeCuNi hybrid composites rather than treating HEA addition as a monotonically beneficial strengthening effect.

## 2. Materials and Methods

### 2.1. Materials and Composite Fabrication Process

Commercially available high-purity copper powder (99.99%, d_50_ ≈ 21 µm; Nanografi, Ankara, Turkey) was employed as the matrix material. The Cu powder was used as received, and the specific industrial powder-production route was not disclosed in the supplier information available to the authors. The AlCrFeCuNi high-entropy alloy (HEA) powder used as the metallic reinforcement had previously been produced by 25 h of mechanical alloying, and its production procedure and detailed structural and mechanical characterization were reported in our previous study [21]. The HEA powder had a median particle size (d50) of approximately 12 µm, whereas the previously reported value of 10.2 nm corresponds to its XRD-derived average crystallite size. This value refers to the internal crystalline domains of the micrometer-scale HEA powder particles and not to their physical particle dimensions. B_4_C powder (d_50_ ≈ 5 µm; Nanografi, Ankara, Turkey) was used as the second reinforcement phase. The B_4_C content was maintained at 1 wt.% in all compositions, whereas the HEA content was varied as 0, 10, 20, and 30 wt.% to examine the effect of reinforcement fraction on the mechanical, electrical, and tribological properties of the Cu–B_4_C composites. The Cu–1 wt.% B_4_C composition without HEA (0HEA) was used as the internal reference for evaluating the effect of increasing HEA content. The B_4_C fraction was intentionally kept constant at 1 wt.% throughout the experimental series so that the HEA fraction remained the principal compositional variable. A Cu-only reference was not included because the present study was designed to investigate the evolution of properties within the B_4_C-containing hybrid system rather than to independently quantify the reinforcing effect of B_4_C. In addition, Cu–AlCrFeCuNi composites without B_4_C had already been investigated in our previous study [23], including an SPS-consolidated Cu–20 wt.% AlCrFeCuNi composition. The present experimental design was therefore selected to extend this previous work toward a B_4_C-containing hybrid architecture while systematically varying the HEA fraction. The compositions and milling parameters are summarized in Table 1.

**Figure 1:**
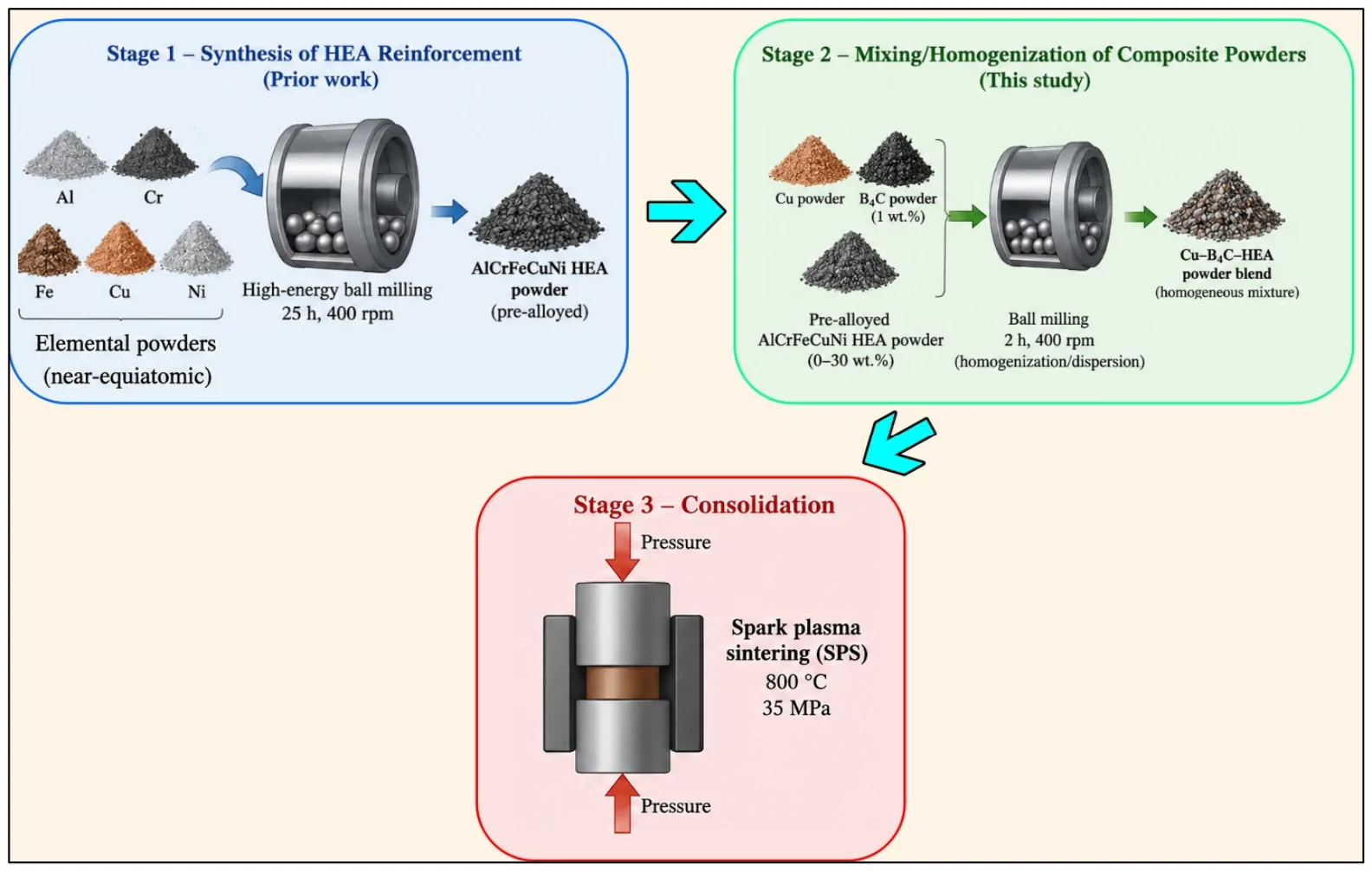
Schematic illustration of the powder-processing route, distinguishing three successive stages: prior synthesis of the nanocrystalline AlCrFeCuNi HEA reinforcement by 25 h mechanical alloying (stage 1), Cu–1 wt.% B_4_C–HEA powder blends for homogenization in the present study (stage 2), and SPS consolidation (stage 3).

The overall powder-processing route is summarized schematically in Figure 1. The processing sequence comprises three clearly separated stages: prior synthesis of the AlCrFeCuNi HEA reinforcement by 25 h of mechanical alloying [21], preparation of the Cu–1 wt.% B_4_C–HEA powder blends by 2 h of milling at 400 rpm in the present study, and subsequent SPS consolidation at 800 °C under 35 MPa. Thus, the first milling stage corresponds to HEA formation, whereas the second is limited to homogenization and dispersion of the pre-alloyed HEA and B_4_C within the Cu-based powder mixture.

To promote a uniform distribution of the constituent powders, the powder blends were mechanically milled in a Retsch PM100 planetary ball mill (Verder company, Düsseldorf, Germany). Milling was conducted for 2 h at 400 rpm using a ball-to-powder weight ratio of 10:1. Methanol was introduced at 2 wt.% as a process control agent to reduce powder sticking and suppress excessive cold welding during milling. The selected milling conditions were established through preliminary experiments, which indicated that lower milling energies resulted in inadequate dispersion, whereas higher energy inputs promoted excessive cold welding.

Following milling, the hybrid powder mixtures were consolidated by spark plasma sintering (SPS) using a Çelmak SPS–160V (Çelmak Makina, Elazığ, Türkiye) system. The powder mixtures were loaded into a graphite die and processed under a nitrogen atmosphere. The samples were heated at a rate of 150 °C min^−1^ to a sintering temperature of 800 °C and held at this temperature for 4 min under a uniaxial pressure of 35 MPa. A pulse on/off ratio of 12:2 was employed. After completion of the SPS cycle, the consolidated specimens were allowed to cool within the SPS chamber under a nitrogen atmosphere. Specimens of approximately 20 mm × 20 mm × 10 mm were subsequently prepared for microstructural, hardness, and tribological characterization, while the specimen geometry used for electrical conductivity measurements is described separately in the corresponding characterization procedure.

### 2.2. Characterization

Following SPS consolidation, the hybrid composite specimens were subjected to conventional metallographic preparation prior to microstructural and phase characterization. Microstructural observations were performed using a Thermo Scientific Apreo 2S (Thermo Fisher Scientific, Eindhoven, The Netherlands) field-emission scanning electron microscope (FE-SEM) coupled with a Thermo Scientific UltraDry (Thermo Fisher Scientific, Eindhoven, The Netherlands) energy-dispersive X-ray spectroscopy (EDS) system. The worn surfaces and deformation features generated during the tribological tests were further examined by SEM to identify the dominant wear mechanisms. Phase identification was conducted by X-ray diffraction (XRD) using a PANalytical X’Pert3 Pro (Bruker, Westborough, MA, USA) diffractometer with Cu Kα radiation (λ = 1.541874 Å). Diffraction patterns were collected over a 2θ range of 30–90° with a step size of 0.01° and a counting time of 1 s per step. The densities of the Cu-based composites were determined according to Archimedes’ principle. Particle size distributions of the powders were determined using a Malvern Mastersizer Hydro 2000e particle size analyzer. Prior to measurement, the powders were dispersed in deionized water and ultrasonically agitated for at least 5 min to minimize particle agglomeration and obtain a homogeneous suspension. Following dispersion, particle size measurements were carried out using the instrument’s computer-controlled analysis software. Brinell hardness measurements were carried out using an Innovatest Nemesis 9000 (Innovatest–Nemesis 9000, Maastricht, The Netherlands) hardness tester under a load of 31.25 kg. Six measurements were taken from different locations on each specimen, and the mean value was reported. The electrical conductivity of the fabricated composites was measured using a portable SIGMASCOPE^®^ SMP10 (Helmut Fischer GmbH, Sindelfingen, Germany) conductivity meter equipped with an ES40 probe, based on the phase-sensitive eddy-current method. Measurements were performed on flat, polished disk-shaped specimens with a diameter of approximately 20 mm and a thickness of approximately 3 mm. These dimensions provided a measurement area sufficiently larger than the effective probe area and a specimen thickness above the minimum required for reliable eddy-current measurements. For each composition, five measurements were taken at different locations on the specimen surface, and the average value was reported as %IACS according to the International Annealed Copper Standard. The specified measurement accuracy of the instrument is approximately ±1% within the 1–100% IACS range at 20 °C.

The tribological performance of the composites was investigated at ambient temperature using a ball-on-disc friction and wear tester equipped with a reciprocating module (UTS Tribolog, Trabzon, Türkiye). A 6 mm diameter 100Cr6 bearing steel ball with a hardness of approximately 60 HRC was employed as the counterface in all experiments. Previous studies on Cu-based metal matrix composites have generally employed light-to-moderate normal loads. For Cu–B_4_C–HEA-type hybrid systems, reported test loads typically range from 2.5 to 20 N, with 1 and 20 N representing the lower and upper limits, respectively [13,23]. Preliminary experiments conducted in the present study showed that loads above 10 N caused severe surface damage, making reliable interpretation of the wear mechanisms difficult. Accordingly, a normal load of 5 N was selected to provide reproducible wear conditions and enable meaningful comparison among the composite specimens. The total sliding distance was maintained at 120 m, corresponding to 5000 reciprocating cycles, for all compositions. The tests were conducted in accordance with ASTM G133 [29] using a stroke length of 12 mm and an oscillation frequency of 3 Hz. Each test condition was repeated three times, and a new 100Cr6 steel ball was used for every measurement to improve statistical reliability. The tribological parameters were intentionally maintained constant for all compositions because the primary objective of the present testing was to compare the effect of HEA content under a common contact condition rather than to establish load- or frequency-dependent wear maps. Accordingly, the tribological results reported in this study should be interpreted specifically within the ASTM G133 reciprocating dry-sliding condition employed here. After the wear tests, the wear-scar depth profiles and volume losses were quantified using a 3D surface profilometer (Nanofocus µscan, Oberhausen, Germany).

## 3. Results and Discussion

### 3.1. Powder Characterization

**Figure 2:**
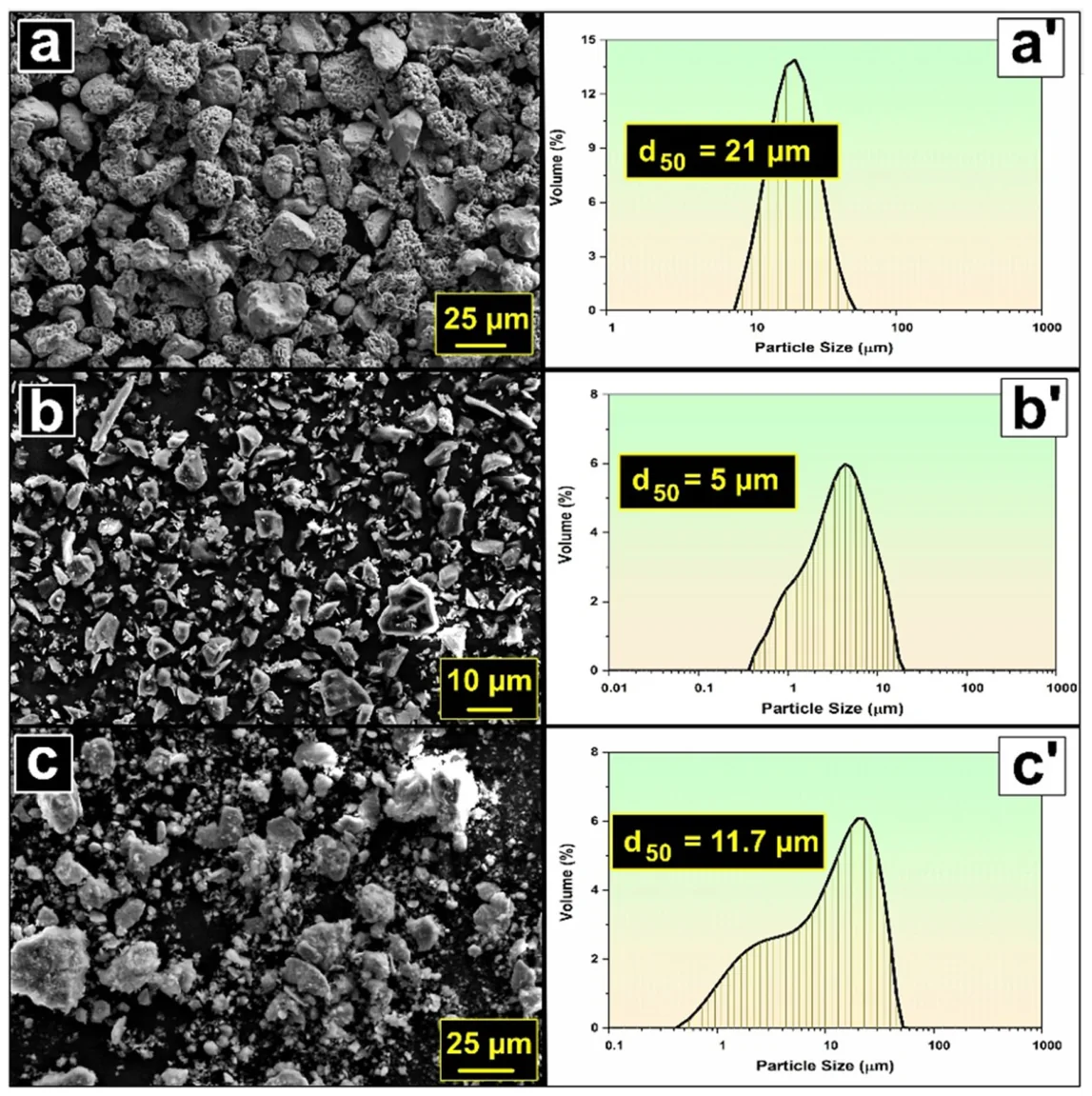
SEM micrographs and corresponding particle size distributions of (**a**,**a′**) as-received Cu powder, (**b**,**b′**) as-received B_4_C powder, and (**c**,**c′**) pre-alloyed nanocrystalline AlCrFeCuNi HEA reinforcement previously synthesized by 25 h mechanical alloying.

Figure 2 shows the SEM micrographs and corresponding particle size distributions of the Cu, B_4_C, and HEA powders used in this study. The as-received Cu powder exhibits a predominantly rounded to near-spherical morphology, although some particles deviate from an ideal spherical geometry (Figure 2a). The corresponding particle size distribution is relatively narrow, indicating a comparatively homogeneous starting powder population. (Figure 2a′). B_4_C particles exhibit an angular morphology with pronounced sharp edges and irregular surfaces (Figure 2b). The corresponding particle size distribution is broader than that of the Cu powder and contains particles of varying sizes (Figure 2b′). The HEA powder, previously subjected to 25 h of mechanical alloying [21], displays a markedly irregular and heterogeneous morphology without a well-defined geometrical shape (Figure 2c). Fine, nearly spherical particles coexist with substantially larger particles exhibiting irregular contours and sharp edges. This morphological heterogeneity is also evident in the particle size distribution (Figure 2c′), which extends over a wide size range and exhibits a distinct bimodal profile. The distribution comprises both submicron-sized particles and relatively coarse particles reaching approximately 70–80 µm.

**Figure 3:**
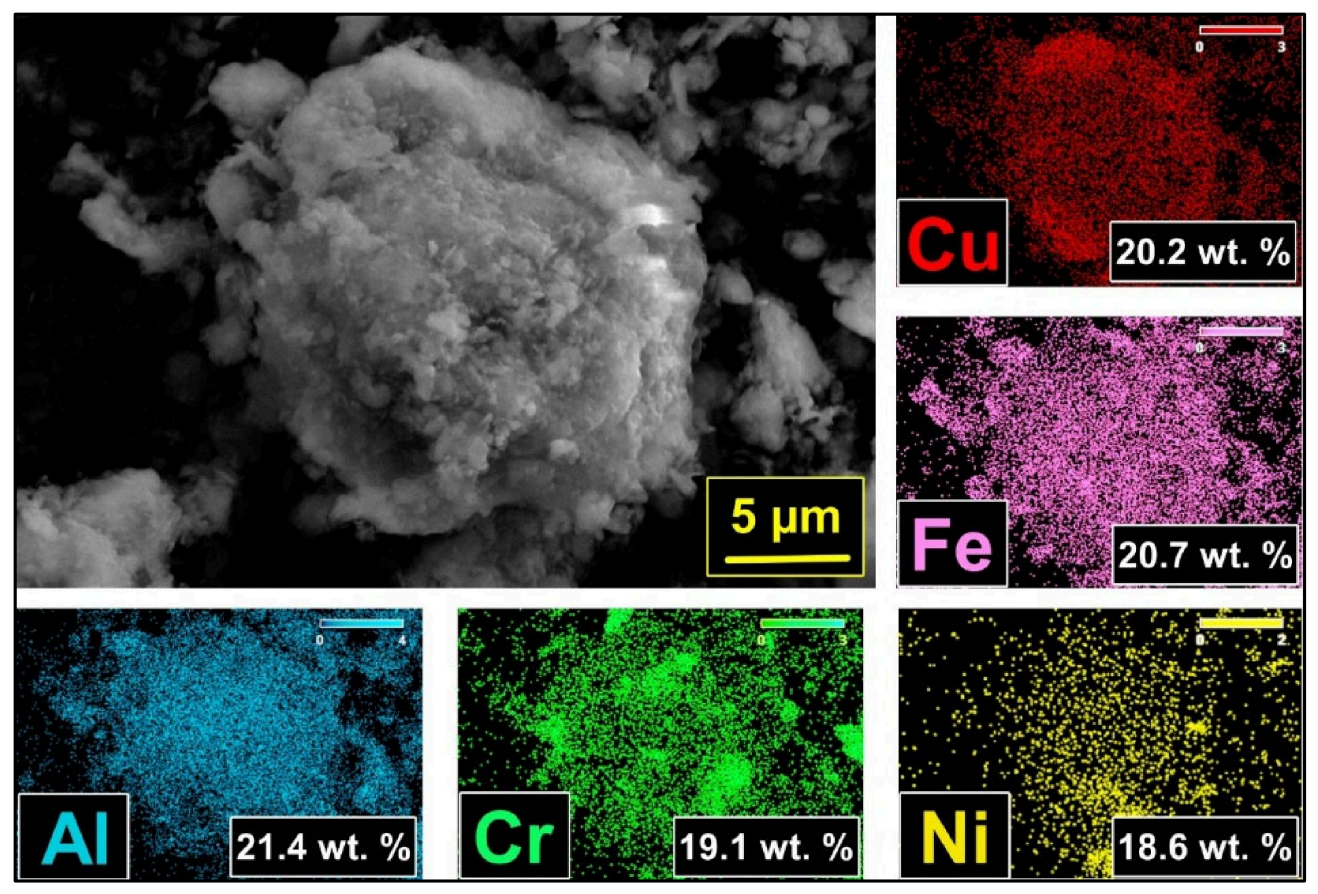
SEM micrograph and corresponding EDS elemental mapping of the AlCrFeCuNi HEA powder after 25 h of mechanical milling.

Figure 3 presents the SEM micrograph and corresponding EDS elemental maps of the AlCrFeCuNi HEA powder after 25 h of mechanical milling. In the elemental maps, Cu, Fe, Al, Cr, and Ni are represented by red, pink, blue, green, and yellow, respectively. The mapping results show that all constituent elements are distributed throughout the analyzed powder particles, with no pronounced elemental segregation or large regions dominated by an individual element. Although slight local variations in elemental intensity can be observed, the overall distribution remains relatively uniform across the examined area. This compositional homogeneity indicates that the prolonged mechanical milling process promoted effective intermixing of the constituent metallic powders and reduced the initial chemical heterogeneity between the individual components. The simultaneous presence and spatially overlapping distribution of Cu, Fe, Al, Cr, and Ni throughout the particles provide evidence of substantial mechanical alloying at the microscale. These observations support the successful formation of a chemically homogeneous multi-principal-element AlCrFeCuNi alloy powder after 25 h of milling.

**Figure 4:**
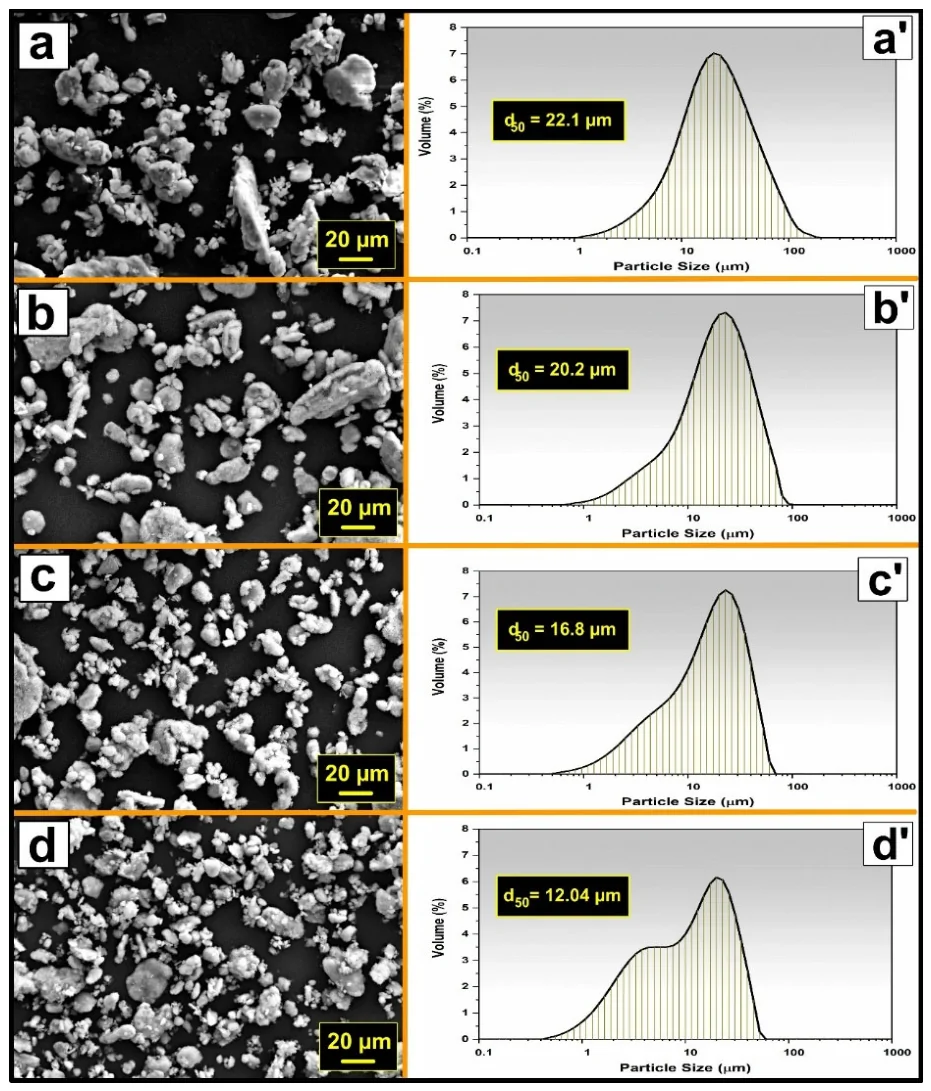
SEM images and particle size distributions of 2 h milled powders: (**a**,**a’**) 0HEA, (**b**,**b’**) 10HEA, (**c**,**c’**) 20HEA and (**d**,**d’**) 30HEA.

Figure 4 presents the SEM micrographs and corresponding particle size distributions of the Cu-based powder mixtures containing 1 wt.% B_4_C and different amounts of AlCrFeCuNi HEA after 2 h of milling. The 0HEA powder mixture exhibits an irregular particle morphology consisting of relatively coarse Cu particles together with finer angular B_4_C particles and small fragmented particles (Figure 4a). The particle size distribution is comparatively broad, with a median particle size (d_50_) of 22.1 µm (Figure 4a′). Although the starting Cu powder exhibits a predominantly rounded to near-spherical morphology, the short milling treatment produces a certain degree of deformation and surface irregularity as a result of repeated particle–ball and particle–particle collisions [30,31]. In the 10HEA powders, the particle morphology remains heterogeneous; however, the fraction of relatively fine particles becomes more pronounced (Figure 4b). The d50 value decreases from 22.1 to 20.2 µm, corresponding to an approximately 8.6% reduction compared with the 0HEA composition (Figure 4b′). This decrease can be associated with the incorporation of the finer, previously milled HEA powder into the Cu–B_4_C mixture and with the fragmentation occurring during the subsequent 2 h milling step. The particle size distribution nevertheless remains relatively broad, indicating the simultaneous presence of coarse Cu-rich particles and finer reinforcement-containing particles. A further increase in the HEA content to 20 wt.% results in a more apparent refinement of the powder mixture (Figure 4c). The number of fine particles increases, while the population of coarse and elongated particles is reduced compared with the 0HEA and 10HEA powders. Consistent with this morphological evolution, the d_50_ value decreases to 16.8 µm (Figure 4c′), representing a reduction of approximately 16.8% relative to the 10HEA composition. The size distribution also shifts toward smaller particle sizes, suggesting that the increasing fraction of the finer and relatively hard HEA particles promotes a greater contribution of fragmentation during milling [22,32]. The most pronounced particle refinement is observed for the 30HEA powder mixture (Figure 4d). The microstructure is dominated by smaller irregular and fragmented particles, whereas the fraction of large particles becomes markedly lower. The corresponding d50 value decreases to 12.04 µm (Figure 4d′), which is approximately 28.3% lower than that of the 20HEA sample and 45.5% lower than that of the 0HEA composition. The particle size curve also develops a broader contribution toward the finer-size region, indicating a more heterogeneous particle population at the highest HEA content. Since the B_4_C content was maintained constant at 1 wt.% in all compositions, the systematic decrease in d50 can primarily be associated with the increasing proportion of the finer HEA powder and its influence on the balance between particle deformation, fragmentation, and cold welding during milling.

**Figure 5:**
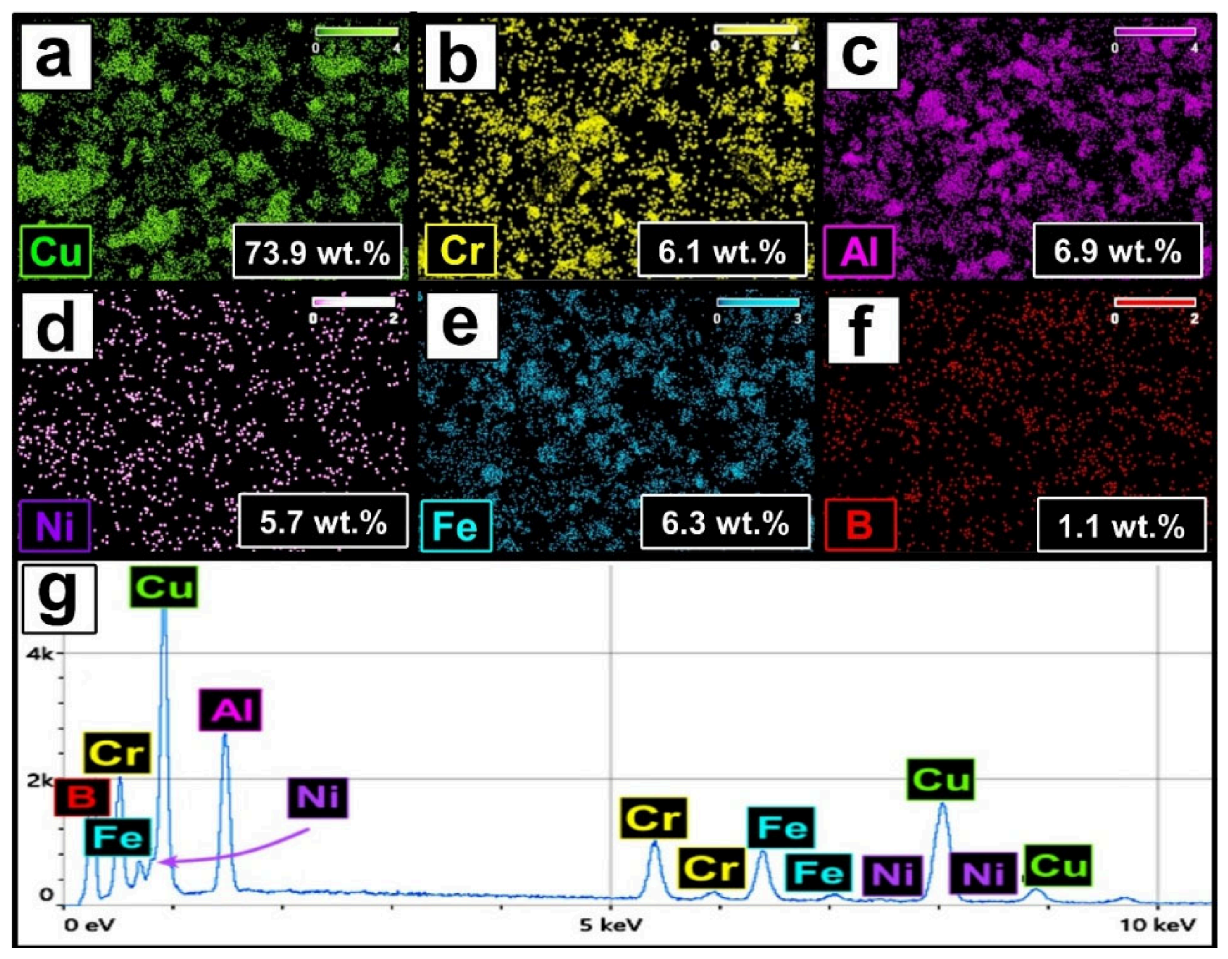
EDS elemental mapping and corresponding spectrum of the 30HEA powder mixture after 2 h of milling: (**a**) Cu, (**b**) Cr, **(c**) Al, (**d**) Ni, **(e**) Fe, (**f**) B, and (**g**) EDS spectrum.

Figure 5 presents the EDS elemental mapping and corresponding spectrum of the 30HEA powder mixture after 2 h of milling. The elemental maps reveal the presence of Cu, Cr, Al, Ni, Fe, and B throughout the analyzed region. Cu constitutes the dominant component, as expected for the Cu-based matrix, whereas Cr, Al, Ni, and Fe exhibit broadly overlapping spatial distributions without pronounced element-rich regions or large-scale segregation. This distribution indicates that the AlCrFeCuNi HEA particles were effectively dispersed within the Cu matrix during milling. Boron, originating from the 1 wt.% B_4_C addition, is detected at a lower intensity but remains distributed across the analyzed area, suggesting that the B_4_C reinforcement was also incorporated throughout the powder mixture rather than being concentrated in isolated regions. The quantitative EDS results are consistent with the nominal composition of the 30HEA sample. Cu accounts for 73.9 wt.% of the detected elements, while Cr, Al, Ni, and Fe are measured as 6.1, 6.9, 5.7, and 6.3 wt.%, respectively. The relatively similar concentrations of these four HEA constituents are consistent with the equal-weight composition used in preparing the AlCrFeCuNi alloy. The comparatively high Cu content arises from the combined contribution of the Cu matrix and the Cu constituent of the HEA reinforcement.

**Figure 6:**
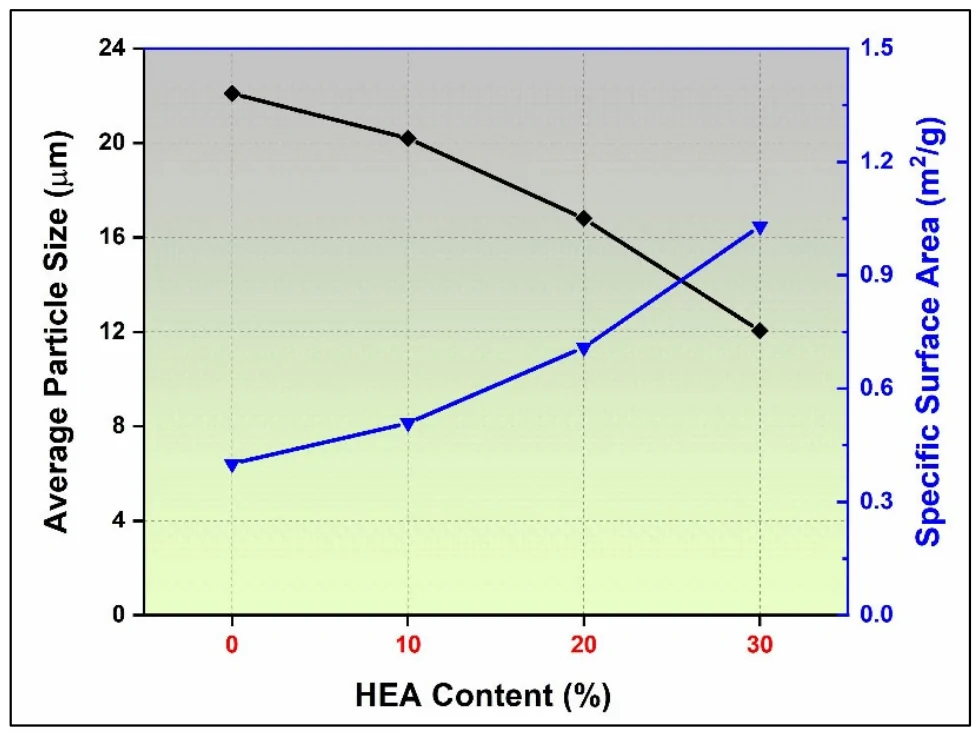
Variation in average particle size and specific surface area of the Cu-B_4_C powder mixtures as a function of HEA content after 2 h of milling.

The evolution of average particle size and specific surface area as a function of HEA content is presented in Figure 6. A clear inverse relationship is observed between these two parameters. The average particle size decreases progressively from approximately 22.1 µm for the 0HEA composition to 20.2, 16.8, and 12.04 µm for the 10HEA, 20HEA, and 30HEA powders, respectively. Thus, increasing the HEA content from 0 to 30 wt.% results in an overall reduction of approximately 45% in particle size. This behavior is consistent with the SEM observations and particle size distributions, which show an increasing fraction of finer particles with increasing HEA content. In contrast, the specific surface area increases continuously with HEA addition, rising from approximately 0.4 m^2^/g for 0HEA to about 0.5, 0.7, and 1.0 m^2^/g for the 10HEA, 20HEA, and 30HEA compositions, respectively. The increase in specific surface area is directly associated with the progressive reduction in particle dimensions, since finer particles provide a greater total surface area per unit mass [33]. The more pronounced increase observed at higher HEA contents is consistent with the enhanced fragmentation of the powder mixture during milling and the resulting decrease in the fraction of coarse particles. The simultaneous decrease in particle size and increase in specific surface area indicate that increasing the HEA fraction substantially modifies the physical characteristics of the powder mixture prior to SPS consolidation. A larger specific surface area increases the number of particle–particle contact regions and may facilitate interparticle diffusion and neck formation during SPS [34,35]. At the same time, the finer particles can occupy voids between larger Cu-rich particles, potentially improving powder packing.

### 3.2. Microstructural Analysis

**Figure 7:**
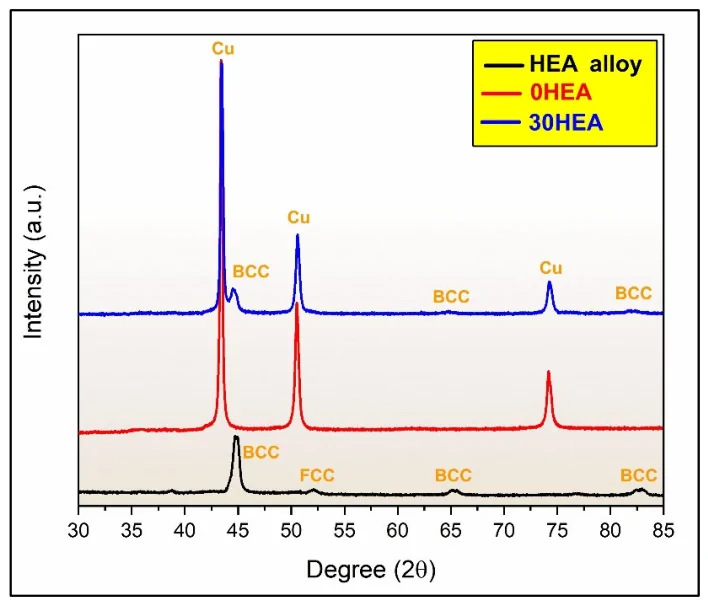
XRD patterns of the pre-alloyed nanocrystalline AlCrFeCuNi HEA reinforcement previously synthesized by 25 h mechanical alloying and the SPS-consolidated 0HEA and 30HEA composites.

Figure 7 presents the XRD patterns of the AlCrFeCuNi HEA powder and the SPS-consolidated 0HEA and 30HEA composites. The 0HEA and 30HEA compositions were selected as representative end members for the post-SPS phase analysis. The 0HEA specimen establishes the phase constitution of the Cu–1 wt.% B_4_C baseline in the absence of HEA, whereas the 30HEA specimen contains the highest HEA fraction and therefore provides the greatest diffraction sensitivity to HEA-related features and to any major crystalline secondary phases that might form during SPS. The present XRD analysis should therefore be interpreted as an end-member comparison rather than as a quantitative assessment of progressive phase evolution across all four HEA contents. The mechanically alloyed HEA exhibits diffraction reflections at approximately 44.5°, 64.6°, and 82.3° 2θ, which are assigned to the BCC structure, together with a weaker reflection at approximately 52.18° corresponding to the FCC phase. The coexistence of these reflections confirms the dual FCC–BCC phase constitution of the HEA powder and is consistent with the detailed structural characterization reported previously for this mechanically alloyed alloy [21]. In that study, XRD-based crystallite-size analysis yielded an average crystallite size of 10.2 nm for the AlCrFeCuNi HEA powder after 25 h of mechanical alloying [21]. This value refers specifically to the crystallite size of the starting HEA powder prior to its incorporation into the Cu–B_4_C matrix. Since crystallite-size analysis was not performed after the subsequent 2 h homogenization milling step or after SPS consolidation, the present XRD results are used only to evaluate phase retention and do not establish whether the initial crystallite size was preserved after processing. The broad HEA reflections observed in the present diffraction pattern are consistent with this previously established nanocrystalline structure. It should be emphasized that the nanoscale dimension refers to the crystalline domains within the powder particles rather than to the particle dimensions themselves. The pronounced diffraction-peak broadening is consistent with the severe plastic deformation and crystallite refinement associated with prolonged mechanical alloying [36]. The coexistence of FCC and BCC phases after extended mechanical milling has also been reported for HEAs with comparable compositions [37,38,39]. The XRD pattern of the 0HEA sample is dominated by three intense reflections located at approximately 43.4°, 50.5°, and 74.2° 2θ, corresponding to the (111), (200), and (220) planes of FCC Cu, respectively (JCPDS card no. #04-0836). The high intensity and relatively sharp character of these peaks indicate that the fundamental FCC crystal structure of the Cu matrix is retained after SPS consolidation. Although the initial composition contains 1 wt.% B_4_C, no distinct diffraction peak attributable to B_4_C can be identified. This behavior can primarily be attributed to the low B_4_C content relative to the Cu matrix. At a reinforcement level of only 1 wt.%, the diffraction intensity generated by B_4_C may be considerably weaker than that of the Cu matrix and may therefore remain below the practical detection limit of conventional XRD analysis [40,41]. The dispersion of B_4_C particles within the matrix and possible overlap of weak B_4_C reflections with the intense Cu peaks or background signal may further reduce their detectability. Therefore, the absence of clearly resolved B_4_C peaks should not be interpreted as evidence for the absence of B_4_C in the composite, but rather as a consequence of its low concentration and limited diffraction contribution under the applied measurement conditions. Similarly, the absence of additional boride- or carbide-related reflections should not be interpreted as definitive evidence for the absence of localized interfacial reactions, since the B_4_C fraction is only 1 wt.% and any reaction products confined to the particle interfaces may be present below the detection capability of conventional XRD. The diffraction pattern of the 30HEA composite is also dominated by the Cu reflections located at approximately 43.4°, 50.5°, and 74.2°, indicating that FCC Cu remains the principal crystalline matrix phase after SPS despite the addition of 30 wt.% HEA. In contrast to the 0HEA sample, however, the 30HEA pattern exhibits an additional weak reflection or shoulder on the higher-angle side of the Cu (111) peak at 44.5°. The position of this feature is consistent with the principal BCC reflection observed in the initial HEA powder. Similarly, weak reflections detected at approximately 64–65° and 82° can also be associated with the BCC phase of the HEA. The detection of these HEA-related features in the 30HEA specimen confirms that HEA-derived crystalline regions remain present after SPS at the highest reinforcement level investigated. Because 30HEA contains the largest amount of HEA, it also provides the most sensitive composition within the present series for identifying any major crystalline reaction products associated with the HEA during consolidation. No additional well-resolved reflections attributable to such secondary phases were detected. This observation suggests that extensive formation of a major crystalline reaction product did not occur under the applied SPS conditions. However, it does not exclude localized or low-volume-fraction interfacial reactions that may remain below the detection capability of conventional XRD. Since XRD patterns were not obtained for the 10HEA and 20HEA specimens, no claim is made regarding progressive diffraction-phase evolution across the complete composition series; the presence and distribution of HEA-rich regions in these intermediate compositions are instead evaluated using the corresponding SEM/EDS observations.

**Figure 8:**
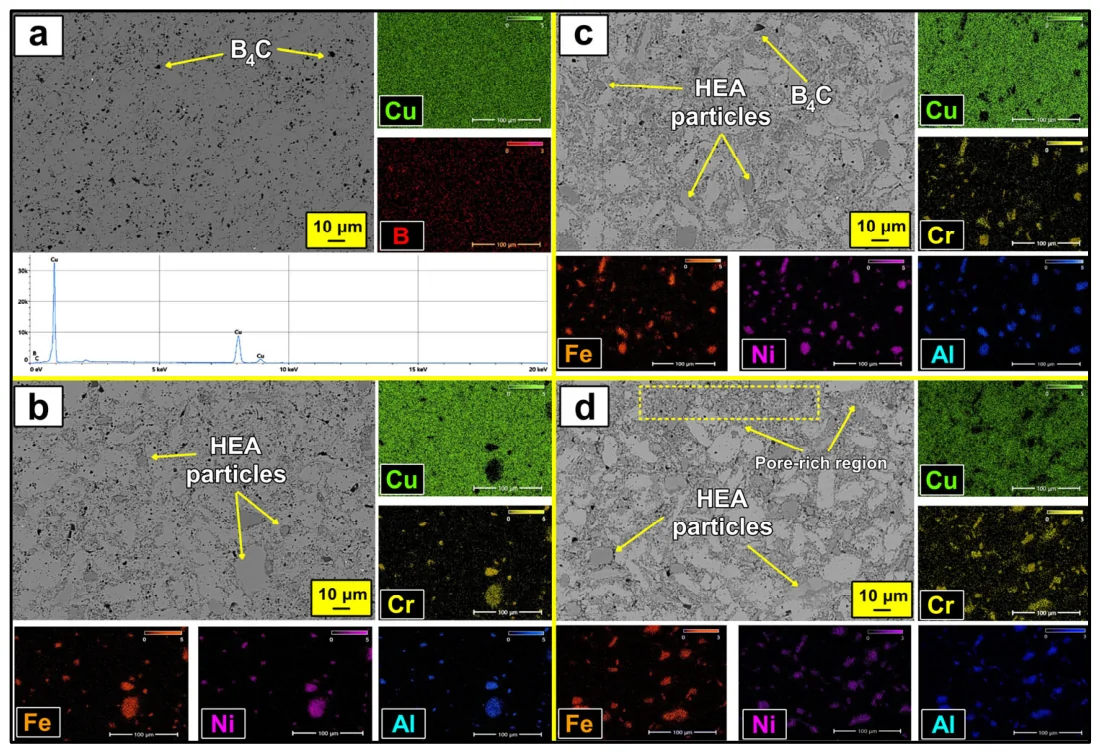
SEM microstructures and corresponding EDS elemental maps of the SPS-consolidated (**a**) 0HEA, (**b**) 10HEA, (**c**) 20HEA, and (**d**) 30HEA composites.

The post-SPS SEM microstructures of the fabricated samples and the corresponding EDS elemental maps are presented in Figure 8. In the elemental maps, Cu, Cr, Fe, Ni, and Al are represented by green, yellow, orange-red, purple, and blue, respectively, while B is shown in red to identify the regions containing B_4_C. As shown in Figure 8a, the microstructure of the 0HEA sample is predominantly composed of the Cu matrix, whereas the dark-contrast regions associated with locally intensified B signals are attributed to B_4_C particles. Because the B_4_C content is limited to only 1 wt.%, the overall intensity of the B signal within the mapped area is relatively low. This observation is consistent with the XRD results, in which no distinct diffraction peak corresponding to B_4_C was detected. At such a low reinforcement content, the diffraction contribution of B_4_C is readily masked by the intense reflections of the Cu matrix, whereas localized B-rich regions can still be distinguished by EDS elemental mapping (Figure 8a). In the HEA-containing samples, the spatial coincidence of Cr, Fe, Ni, and Al signals within the same microstructural regions confirms that the particles exhibiting a different contrast in the SEM images correspond to the AlCrFeCuNi HEA phase. The locally lower Cu intensity within these regions should not be interpreted as the complete absence of Cu; rather, it indicates that the Cu concentration is lower than that in the surrounding matrix. The strong spatial correlation among the Cr, Fe, Ni, and Al maps further indicates that these elements are not randomly distributed as independent phases within the Cu matrix but are predominantly retained together within the HEA particles. In contrast, the Cu signal remains continuous throughout the matrix. When considered together with the XRD results (Figure 7), particularly the retention of the characteristic BCC reflections of the HEA in the 30HEA sample, the EDS observations support the conclusion that the HEA particles remain present as distinct reinforcement regions within the Cu matrix after SPS consolidation.

As shown in Figure 8b, the HEA particles in the 10HEA sample are distributed relatively homogeneously throughout the Cu matrix and remain largely separated from one another. No extensive agglomerated regions are observed, and the continuity of the Cu matrix is largely preserved. An HEA content of 10 wt.% appears to retain a sufficient volume fraction of the ductile Cu matrix to accommodate particle rearrangement and plastic deformation during SPS. Consequently, the Cu particles can deform and fill the spaces surrounding the HEA particles, thereby maintaining effective reinforcement/matrix contact and promoting local densification. Increasing the HEA content to 20 wt.% leads to a pronounced increase in both the number and the area fraction of HEA-rich regions (Figure 8c). The Cr, Fe, Ni, and Al maps indicate that the reinforcement remains distributed throughout the composite; however, the average distance between neighboring HEA particles decreases compared with the 10HEA sample, and some particles begin to approach one another. Although this evolution is expected as the reinforcement fraction increases, it also suggests the onset of a microstructural tendency toward less uniform dispersion at higher HEA contents. Nevertheless, the Cu matrix remains largely continuous in the 20HEA sample, and a substantial fraction of the HEA particles is still surrounded by the matrix.

A more pronounced microstructural change is observed in the 30HEA sample (Figure 8d). The fraction of HEA-rich regions increases substantially, and the HEA particles are positioned much closer to one another in several areas, resulting in localized clustering. This behavior can be related to the reduction in the relative amount of Cu matrix and the corresponding decrease in the average interparticle spacing between HEA particles. The SEM image also reveals pore-rich regions, particularly in areas where HEA particles are closely packed. The preferential occurrence of pores in HEA-rich regions suggests a direct relationship between increasing HEA content and the densification behavior of the composite. This trend can be rationalized by considering the differences in deformation behavior between the Cu matrix and the HEA reinforcement. During SPS, the comparatively deformable Cu matrix can undergo particle rearrangement and localized plastic deformation under the applied pressure, thereby promoting the closure of interparticle voids and densification [42,43]. As the fraction of the harder HEA particles increases, the amount of deformable Cu available to accommodate particle rearrangement decreases, which can restrict densification, particularly in HEA-rich regions. Under these conditions, the ability of the Cu matrix to fill the initial voids between powder particles becomes progressively restricted. When HEA particles are locally agglomerated, penetration and plastic flow of Cu into the interparticle spaces become even more difficult. Consequently, some of the initial interparticle voids may not be completely eliminated during SPS and remain in the final microstructure as residual porosity. Therefore, the increased porosity observed in the 30HEA sample can be attributed not merely to the higher reinforcement fraction but more specifically to HEA agglomeration and the resulting reduction in local densification efficiency.

**Figure 9:**
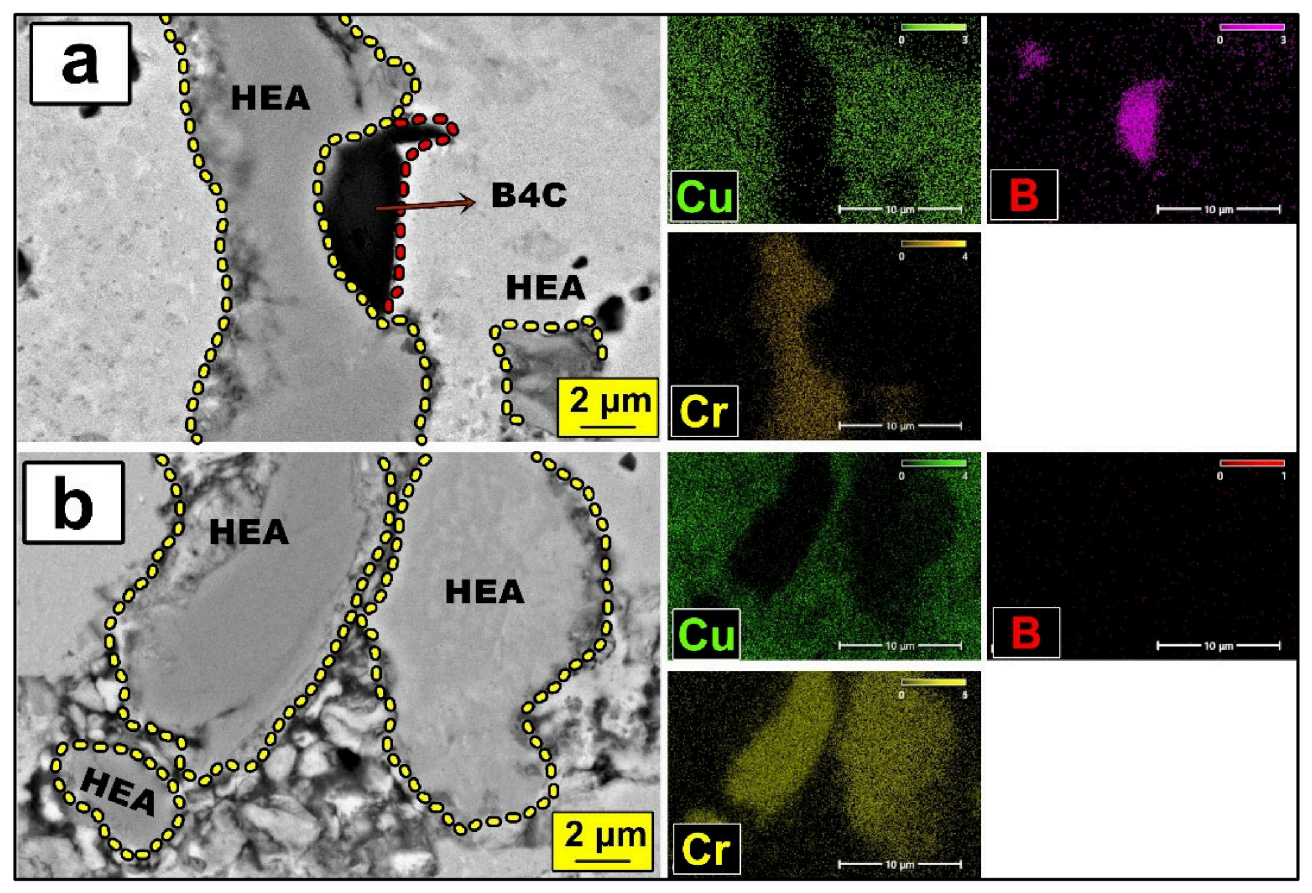
High-magnification SEM images and corresponding EDS elemental maps of the (**a**) 10HEA and (**b**) 30HEA composites.

The higher-magnification SEM images and EDS maps presented in Figure 9 further support these observations and allow a more detailed evaluation of the reinforcement/matrix interfaces in the 10HEA and 30HEA samples. In the EDS analysis, Cr was selected as the representative element for identifying the spatial distribution of the HEA phase. In the 10HEA sample, the boundaries of the HEA particles are clearly distinguishable, while the interface between the HEA particles and the Cu matrix appears continuous and free of pronounced interfacial gaps (Figure 9a). The absence of visible interfacial separation, cracks, or large voids indicates intimate physical contact between the Cu matrix and the HEA particles and is consistent with effective consolidation at the microscale. The enrichment of the Cr signal within the HEA regions and the predominance of the Cu signal in the surrounding matrix further confirm the chemical distinction between the reinforcement and matrix regions. The high-magnification image of the 10HEA sample also reveals a B_4_C particle in close contact with both the HEA phase and the Cu matrix. The localized enrichment of B at the position of the dark-contrast particle supports its identification as B_4_C. No pronounced gap or interfacial separation is observed around the B_4_C particle, indicating good physical contact between the reinforcement and the surrounding phases after consolidation. Such a continuous interface can be associated with the applied pressure and the plastic flow of the Cu matrix during SPS. The plastically deforming Cu can accommodate the comparatively rigid B_4_C and HEA particles and fill the surrounding interparticle spaces, thereby promoting the formation of a more compact and continuous interface. In contrast, the high-magnification image of the 30HEA sample shows that the continuity of the Cu matrix is locally interrupted in regions where HEA particles are positioned in close proximity, and residual voids remain between neighboring particles (Figure 9b). The broad and closely spaced Cr-rich regions in the EDS map further support the presence of HEA agglomeration. At this high reinforcement level, the reduced Cu fraction limits the ability of the matrix to penetrate the narrow spaces between adjacent HEA particles and to accommodate complete interparticle closure during SPS. As a result, not all particle contact regions are effectively densified, leading to the retention of interparticle porosity. Thus, the continuous and nearly pore-free reinforcement/matrix interfaces observed at the lower HEA content progressively evolve into a microstructure characterized by localized HEA agglomeration and more pronounced residual porosity when the HEA content is increased to 30 wt.%.

Although the continuous B_4_C/Cu and B_4_C/HEA contacts observed in Figure 9 are consistent with pressure-assisted consolidation, mechanical interlocking, and plastic accommodation of the Cu matrix, a contribution from localized interfacial reactions cannot be completely excluded. In particular, Cr and Fe within the AlCrFeCuNi reinforcement have a strong chemical affinity for B and C, and intimate particle contact produced during the preceding milling step may favor local interfacial reactions during SPS at 800 °C. Nevertheless, no distinct reaction layer or secondary boride/carbide phase was resolved by the present SEM/EDS and XRD analyses. Considering the low B_4_C content of 1 wt.%, the short SPS holding time of 4 min, and the potentially small volume fraction of any interfacial reaction products, minor Cr- or Fe-containing borides/carbides may remain below the detection capability of the employed characterization methods. Therefore, the present results support predominantly physical and mechanically assisted interfacial contact, while a localized chemical contribution to the interfacial bonding cannot be excluded. Confirmation of such reaction layers would require higher-resolution interfacial characterization.

### 3.3. Relative Density

**Figure 10:**
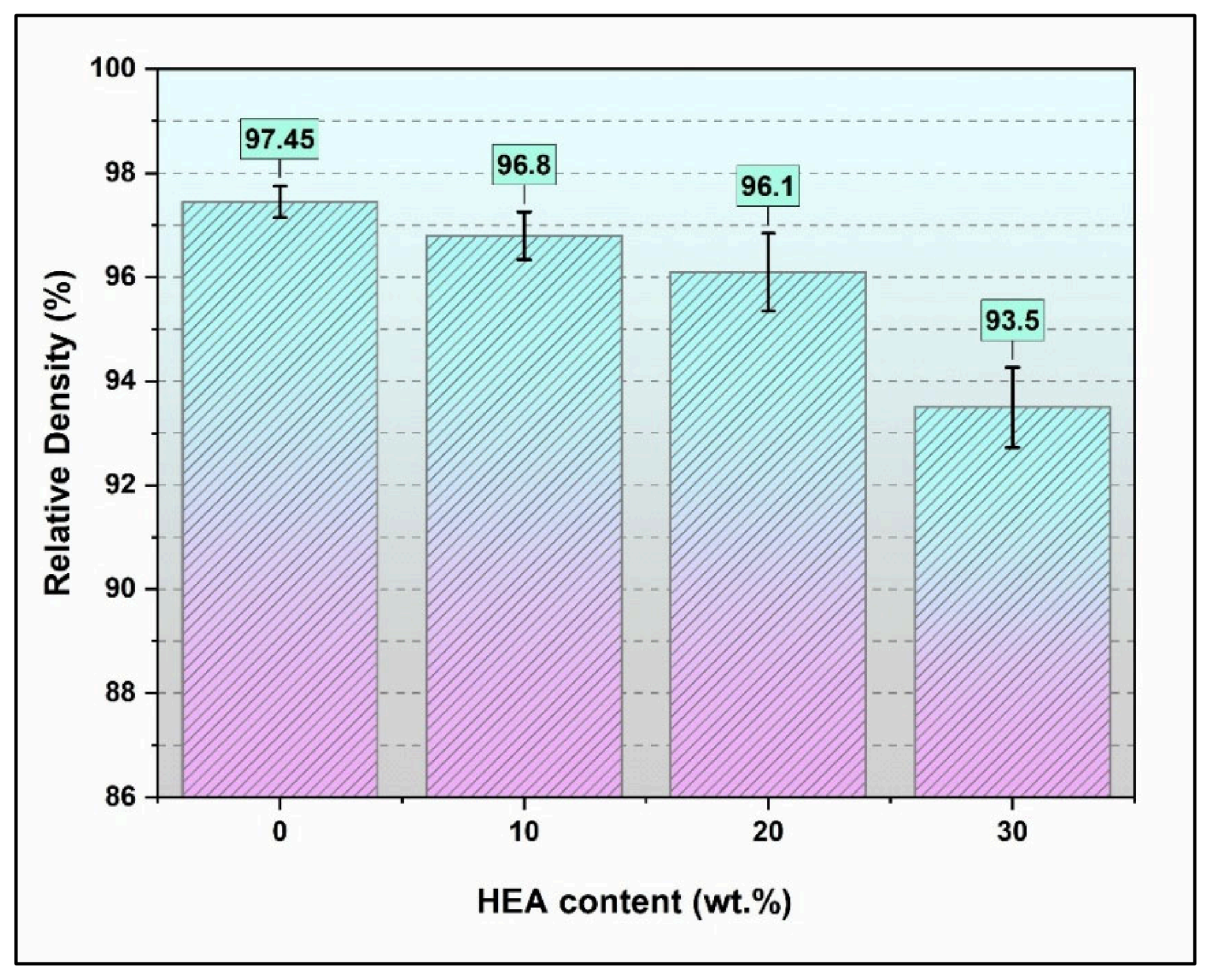
Relative density of the SPS-consolidated Cu–1 wt.% B_4_C composites as a function of AlCrFeCuNi HEA content.

Figure 10 shows the relative densities of the SPS-consolidated 0HEA, 10HEA, 20HEA, and 30HEA composites. The highest relative density was obtained for the 0HEA sample at 97.45%. With increasing HEA content, the relative density decreased progressively to 96.8%, 96.1%, and 93.5% for the 10HEA, 20HEA, and 30HEA samples, respectively. The reduction was relatively limited up to 20 wt.% HEA, whereas a more pronounced decrease occurred between 20HEA and 30HEA, indicating that densification became increasingly restricted at the highest reinforcement level. The density trend is consistent with the SEM and EDS observations (Figure 8 and Figure 9). In the 10HEA and 20HEA samples, the relatively uniform HEA distribution and continuous Cu matrix facilitate plastic deformation and pore closure during SPS, maintaining relative densities above 96%. HEA agglomeration and increased HEA–HEA contacts in the 30HEA sample restrict Cu flow and hinder complete densification, leading to higher residual porosity. Interestingly, the reduction in relative density occurs despite the decrease in median powder size from 22.1 µm for 0HEA to 12.04 µm for 30HEA (Figure 4 and Figure 6). Although finer powders may generally promote sintering by increasing the available contact area, this effect is apparently outweighed at high HEA contents by particle agglomeration, reduced matrix continuity, and the increasing fraction of relatively rigid reinforcement particles. Thus, the densification behavior of the present composites is governed not simply by particle size but by the combined effects of reinforcement fraction, spatial distribution, matrix deformability, and local particle packing.

A similar tendency has been reported for other particle-reinforced Cu-matrix composites. Shu et al. [14] observed a progressive reduction in relative density from approximately 99.1% for unreinforced Cu to 92.3% for a Cu composite containing 20 wt.% B_4_C, which was associated with the increasing fraction of hard ceramic particles and the resulting difficulty in achieving complete densification. Altinsoy et al. [44] reported relative densities ranging from 97.5% to 90.19% for Cu–B_4_C composites containing up to 3 wt.% B_4_C produced by conventional powder metallurgy, further demonstrating the detrimental effect of increasing second-phase content on densification. A particularly relevant comparison is provided by Yanar et al. [23], who investigated Cu–20 wt.% AlCrFeCuNi HEA composites containing graphene under comparable SPS conditions. The relative density decreased from 97.51% for the Cu–HEA composite to 96.84%, 96.13%, and 94.92% with the addition of 0.5, 1.0, and 2.0 wt.% graphene, respectively. The relative densities obtained in the present study therefore fall within the range reported for particle-reinforced Cu composites. More importantly, relative densities above 96% were maintained up to 20 wt.% HEA, indicating effective consolidation within this composition range.

### 3.4. Hardness

**Figure 11:**
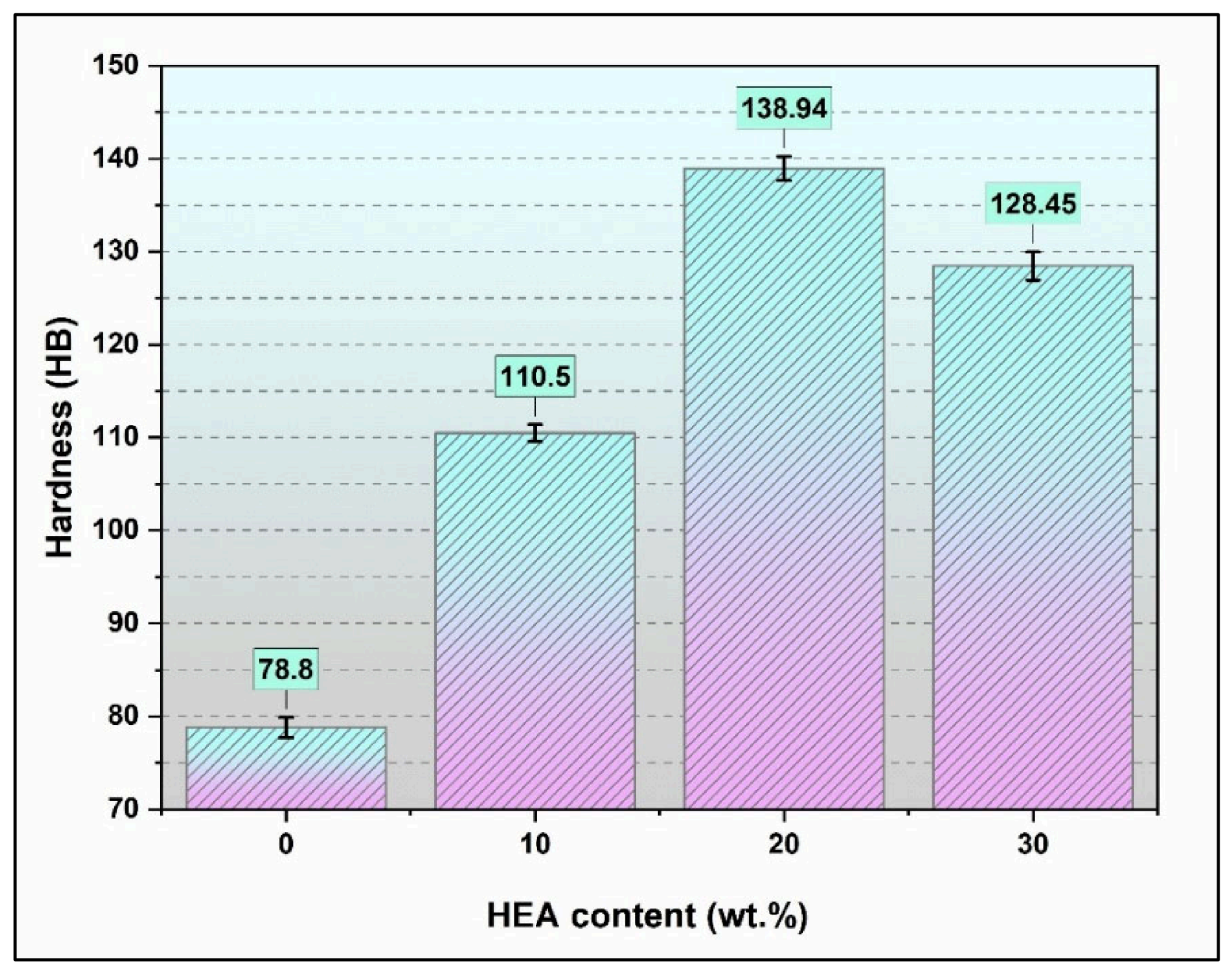
Hardness values of the SPS-consolidated Cu–1 wt.% B_4_C composites as a function of AlCrFeCuNi HEA content.

Figure 11 shows the effect of HEA content on the Brinell hardness of the composites. The hardness increased from 78.8 HB for the 0HEA sample to 110.5 and 138.94 HB with the addition of 10 and 20 wt.% HEA, respectively. Further increasing the HEA content to 30 wt.% reduced the hardness to 128.45 HB; nevertheless, this value remained approximately 63% higher than that of the 0HEA sample. The progressive increase in hardness up to 20 wt.% HEA can primarily be attributed to the increasing fraction of the hard mechanically alloyed HEA reinforcement, which provides greater resistance to plastic deformation than the Cu matrix. The starting HEA powder was previously characterized by an average crystallite size of 10.2 nm [21]. This nanocrystalline internal structure may contribute to the high intrinsic resistance of the reinforcement to deformation; however, the present results do not isolate crystallite-size strengthening from the effects of composition, phase constitution, reinforcement fraction, and interfacial constraint. The relatively uniform distribution of the HEA particles and the preserved continuity of the Cu matrix observed by SEM/EDS, particularly in the 20HEA sample, are favorable for effective load transfer between the matrix and reinforcement. The HEA particles may also impede dislocation motion and constrain local plastic flow within the Cu matrix, thereby increasing resistance to indentation [45]. The decrease in hardness from 138.94 HB for 20HEA to 128.45 HB for 30HEA, despite the higher reinforcement content, indicates that the strengthening effect of the additional HEA is partially offset by microstructural defects at the highest HEA fraction. This interpretation is consistent with the decrease in relative density to 93.5% and the pronounced HEA agglomeration and interparticle porosity observed in the SEM micrographs of the 30HEA sample. The increased porosity reduces the effective load-bearing area, while localized HEA agglomeration disrupts uniform stress transfer between the matrix and reinforcement.

The present trend is consistent with previous studies on HEA-reinforced Cu-matrix composites. Mane et al. [46] reported that the addition of 10 wt.% FeCoNiMnCr HEA increased the microhardness of Cu from 49.2 to 71.1 HV, corresponding to an improvement of approximately 40%, which is comparable to the 40.2% increase obtained for the 10HEA sample in the present study. Zhu et al. [19] similarly reported a 69.8% increase in microhardness for FeCoNiCrAl HEA-reinforced Cu composites produced by friction stir processing, accompanied by Cu-grain refinement and the development of a well-bonded HEA/Cu interfacial region. Although the hardness values obtained using Brinell, Vickers, and nanoindentation methods should not be compared directly on an absolute basis, the reported trends consistently demonstrate the strengthening contribution of HEA particles. Zhang et al. [1], for example, measured local hardness values of 7.5–8.5 GPa within FeCoCrNi HEA particles, approximately 6.0 GPa at the HEA/Cu interface, and about 2.5 GPa in the Cu matrix, confirming a pronounced local resistance to deformation associated with the HEA reinforcement. In the present study, the maximum hardness increase of 76.3% at 20 wt.% HEA therefore reflects a favorable balance between reinforcement content, particle distribution, interfacial integrity, and densification, whereas the subsequent decrease at 30 wt.% HEA is consistent with the detrimental effects of agglomeration and residual porosity.

### 3.5. Electrical Conductivity

**Figure 12:**
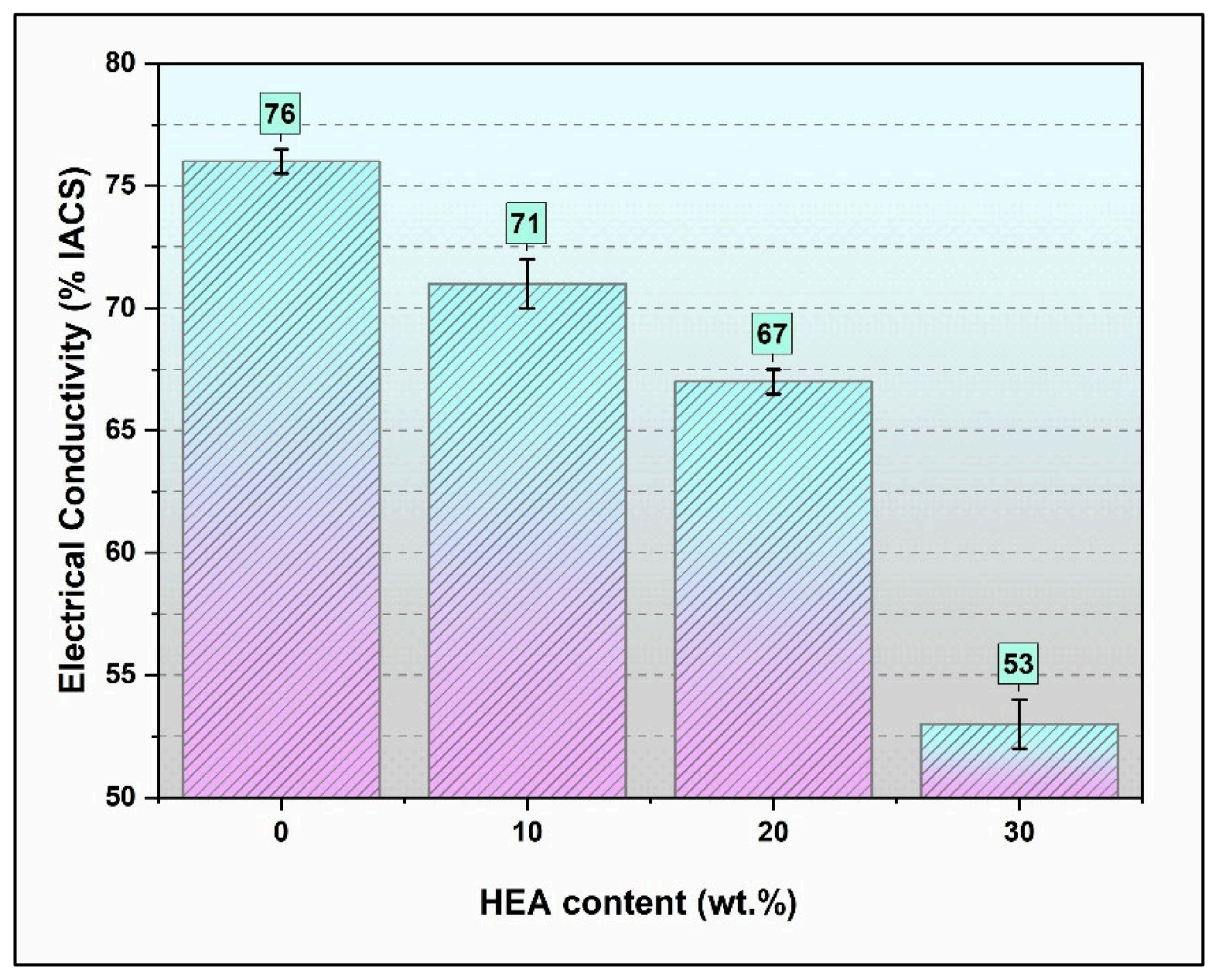
Electrical conductivity values of the SPS-consolidated Cu–B_4_C composites as a function of AlCrFeCuNi HEA content.

Figure 12 shows that the electrical conductivity of the composites decreases progressively with increasing HEA content, from 76% IACS for the 0HEA sample to 71, 67, and 53% IACS for the 10HEA, 20HEA, and 30HEA samples, respectively. This trend can primarily be attributed to the gradual reduction in the fraction and continuity of the highly conductive Cu matrix as the HEA content increases. At the same time, the increasing number of Cu/HEA interfaces introduces additional electron-scattering sites, thereby shortening the effective electron mean free path and increasing electrical resistivity. Up to 20 wt.% HEA, the decrease in conductivity remains relatively moderate, which is consistent with the SEM/EDS observations showing that the Cu matrix largely preserves its continuity and the HEA particles remain reasonably well dispersed (Figure 8). The more pronounced decrease observed for the 30HEA sample is consistent with the development of HEA-rich agglomerates, increased residual porosity, and reduced matrix continuity. These microstructural discontinuities hinder the formation of continuous Cu-rich electron-transport pathways and enhance scattering at interfaces, pores, and structural defects. Thus, the conductivity behavior reflects the combined effects of decreasing Cu fraction, increasing interfacial density, and the progressive deterioration of microstructural continuity at high HEA contents. Despite the reduction in conductivity, the 20HEA sample retains 67% IACS while exhibiting the highest hardness, indicating that this composition provides the most favorable balance between mechanical strengthening and electrical conductivity among the investigated samples.

### 3.6. Tribological Characterization

**Figure 13:**
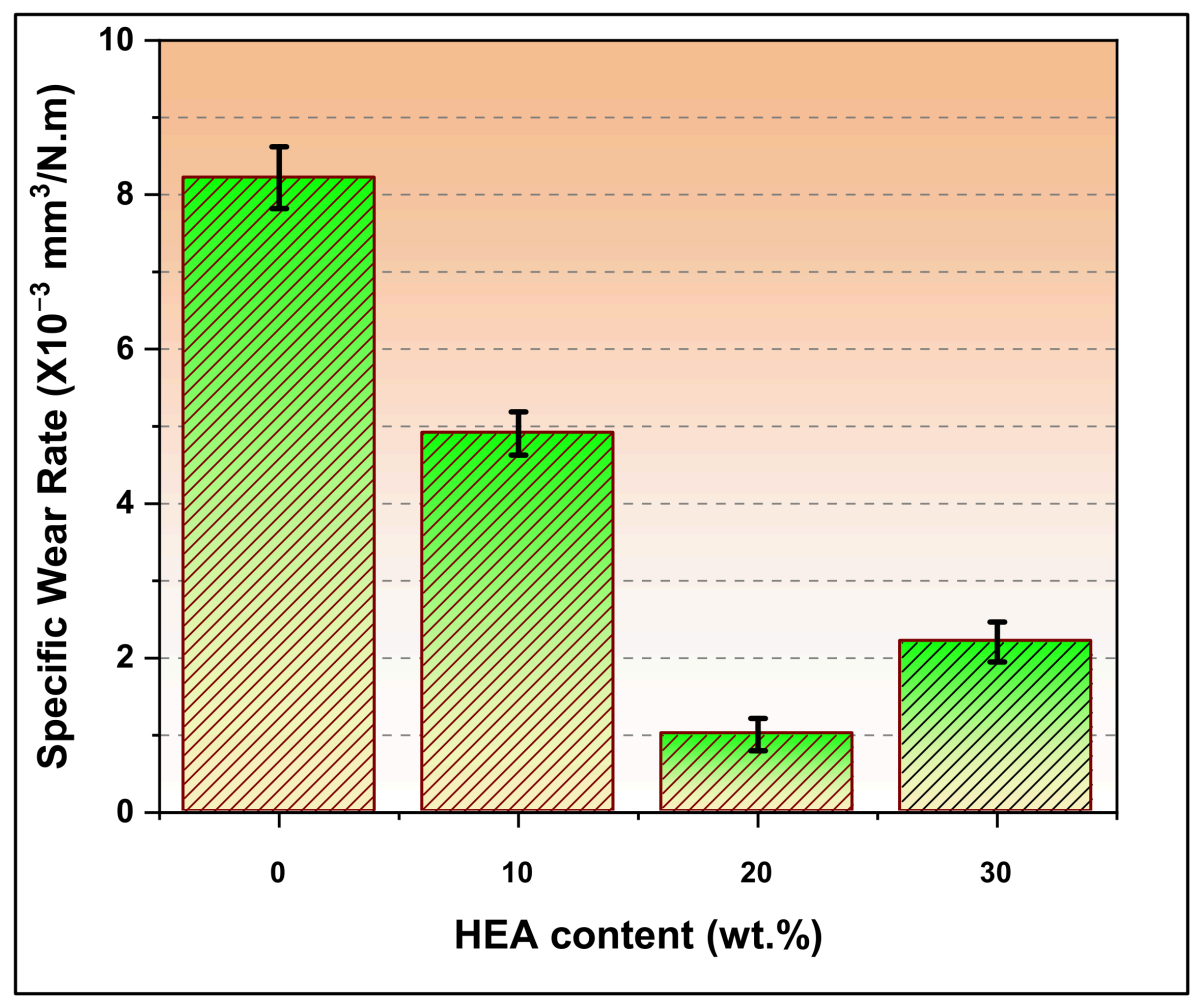
Specific wear rate of the Cu-B_4_C composites as a function of AlCrFeCuNi HEA content.

Figure 13 illustrates that the tribological response of the Cu–B_4_C composites strongly depended on the AlCrFeCuNi HEA content. Under the reciprocating dry-sliding conditions employed in this study, the specific wear rate decreased markedly with increasing HEA content up to 20 wt.%, followed by a partial increase at 30 wt.% HEA. The 0HEA composite exhibited the highest specific wear rate, reaching 8.22 × 10^−3^ mm^3^/N.m. The addition of 10 wt.% HEA reduced this value to 4.91 × 10^−3^ mm^3^/N.m, corresponding to a reduction of about 40% relative to 0HEA. The lowest wear rate, 1.01 × 10^−3^ mm^3^/N.m, was obtained for the 20HEA sample. This represents an approximately 88% reduction compared with 0HEA. Increasing the HEA fraction further to 30 wt.% increased the specific wear rate to approximately 2.21 × 10^−3^ mm^3^/N.m. Although the wear rate of 30HEA remained substantially lower than that of 0HEA and 10HEA, it was more than twice that measured for 20HEA. This behavior can be rationalized by considering the combined effects of reinforcement hardness, reinforcement/matrix contact condition, and densification. As previously reported for the mechanically alloyed AlCrFeCuNi HEA used as the reinforcement [21], the alloy exhibits a microhardness of approximately 500 HV and an average crystallite size of 10.2 nm. Its nanocrystalline internal structure may contribute to the high deformation resistance of the reinforcement, although its individual contribution to the composite wear response cannot be separated from those of HEA composition, phase constitution, reinforcement fraction, interfacial bonding, and densification in the present experiments. Accordingly, the progressive incorporation of the hard HEA particles increases the resistance of the composite surface to localized plastic deformation, counterface penetration, and subsurface shearing during repeated sliding. This strengthening effect is reflected in the increase in bulk hardness from 78.8 HB for 0HEA to 110.5 HB for 10HEA and 138.94 HB for 20HEA (Figure 11). The tribological improvement observed up to 20 wt.% HEA therefore cannot be attributed solely to the increase in reinforcement fraction, but also to the effective mechanical support provided by the hard HEA particles within the comparatively ductile Cu matrix. Another factor that may contribute to this behavior is the Cu-containing nature of the AlCrFeCuNi reinforcement. Because Cu is present in both the matrix and the HEA phase, improved chemical compatibility at the matrix/reinforcement interface may facilitate intimate interfacial contact during SPS consolidation and reduce the tendency for interfacial separation under cyclic shear loading. This interpretation is consistent with the SEM observations showing continuous HEA/Cu interfaces and the absence of pronounced interfacial gaps at lower and intermediate HEA contents (Figure 8). In the 20HEA composite, the hard mechanically alloyed HEA reinforcement is present at a sufficiently high fraction to restrict plastic flow and support the sliding load, while the Cu matrix remains largely continuous and the relative density remains high at 96.1%. This balance provides a mechanically stable load-bearing microstructure without the pronounced clustering and porosity observed at 30 wt.% HEA. The superior performance of 20HEA should therefore not be interpreted as a simple consequence of increasing the amount of nanocrystalline reinforcement; rather, it reflects a balance between reinforcement-induced strengthening, matrix continuity, interfacial support, and densification. When the HEA content is increased to 30 wt.%, however, the composite hardness decreases to 128.45 HB despite the higher fraction of the intrinsically hard HEA phase. This reduction coincides with a decrease in relative density to 93.5%, localized HEA clustering, increased HEA–HEA contacts, and greater residual porosity. Under reciprocating sliding, pores and poorly supported HEA-rich regions can promote local stress concentration, crack initiation, and detachment of reinforcement-containing fragments, while the reduced continuity of the Cu matrix limits uniform stress redistribution around the hard particles.

The deterioration observed when the HEA content is increased from 20 to 30 wt.% is better interpreted by considering the simultaneous evolution of several microstructural and mechanical parameters rather than the HEA fraction alone. Between these two compositions, the relative density decreases from 96.1 to 93.5%, corresponding to an increase in estimated porosity from approximately 3.9 to 6.5%, while the hardness decreases from 138.94 to 128.45 HB. Over the same interval, the specific wear rate increases from 1.01 × 10^−3^ to 2.21 × 10^−3^ mm^3^/N·m, representing an increase of approximately 119%. This deterioration occurs despite the further reduction in median powder size from 16.8 to 12.04 µm. Therefore, powder refinement alone does not account for the tribological response. Up to 20 wt.% HEA, the strengthening contribution of the hard reinforcement is accompanied by relatively high densification and preservation of Cu-matrix continuity. At 30 wt.% HEA, the higher residual porosity, local HEA clustering, increased HEA–HEA contacts, and reduced continuity of the deformable Cu matrix coincide with the decrease in bulk hardness and the increase in wear. The non-monotonic wear behavior therefore reflects the coupled effects of reinforcement-induced strengthening, densification, and reinforcement distribution rather than a direct effect of HEA content or chemistry alone.

**Figure 14:**
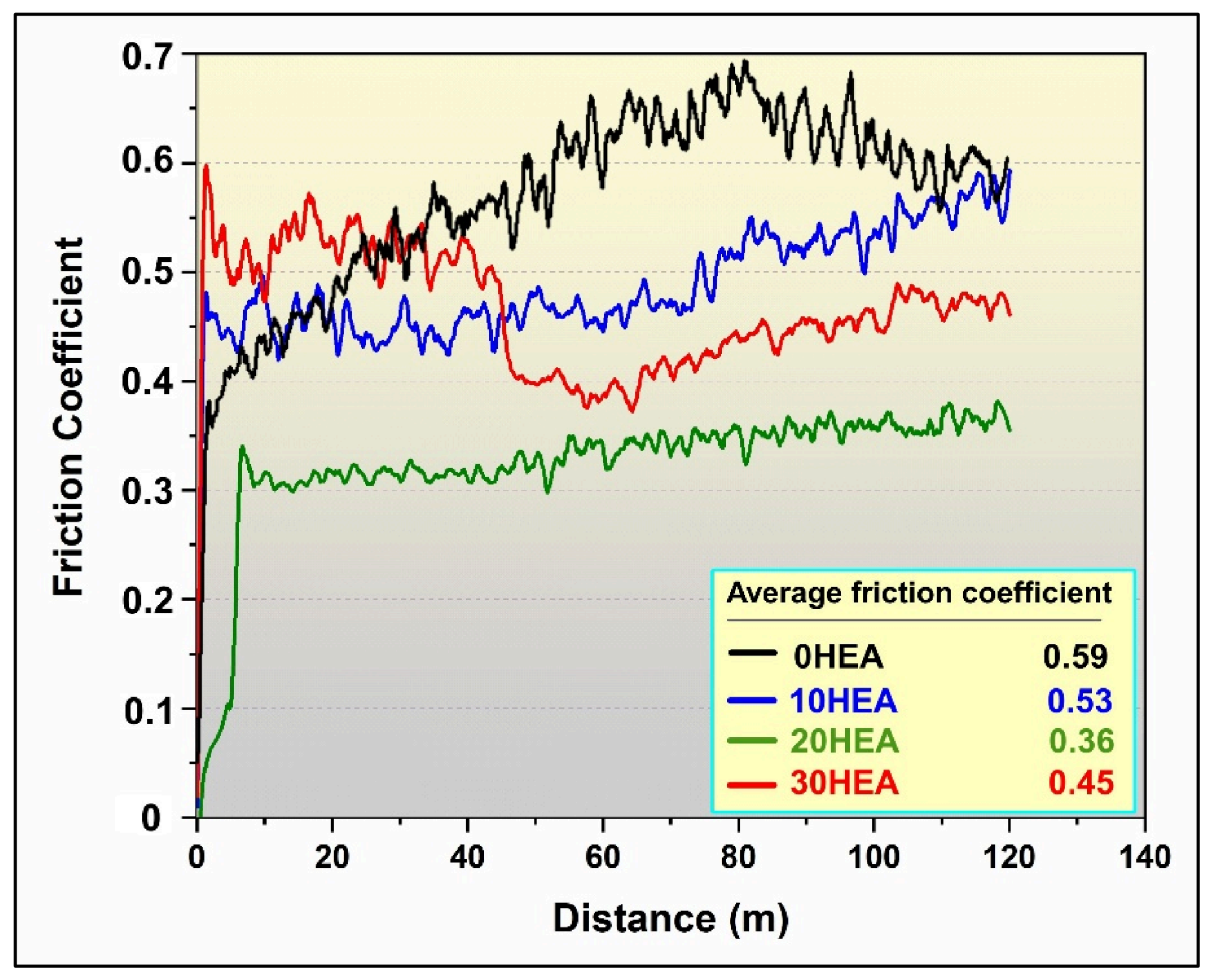
Friction coefficient as a function of sliding distance for the 0HEA, 10HEA, 20HEA, and 30HEA composites.

The friction coefficient curves presented in Figure 14 show that the addition of AlCrFeCuNi HEA affects not only the magnitude of friction but also the evolution and stability of the sliding contact. The average coefficients of friction were determined as 0.59, 0.53, 0.36, and 0.45 for the 0HEA, 10HEA, 20HEA, and 30HEA composites, respectively. Among the investigated compositions, the 0HEA sample exhibits the most pronounced distance-dependent variation. Following the initial running-in period, the friction coefficient gradually increases from approximately 0.35–0.40 and reaches a maximum of about 0.65–0.69 at approximately 75–80 m. This progressive increase is consistent with the behavior expected for a relatively soft Cu-rich matrix subjected to repeated reciprocating contact. Progressive plastic deformation of surface asperities, Cu smearing, enlargement of the real contact area, and repeated formation of adhesive junctions with the 100Cr6 counterface may increase the resistance to sliding. Similar plastic deformation and material-transfer processes have been reported for Cu-based sliding systems, particularly when a relatively soft Cu matrix is brought into contact with a harder counterface [47,48]. The subsequent decrease in friction after approximately 80 m indicates an evolution of the sliding interface after prolonged deformation. The friction behavior of 10HEA is comparatively more regular during the first half of the test. The friction coefficient remains predominantly within the range of approximately 0.43–0.48, although relatively sharp local peaks occur throughout sliding. Beyond approximately 70–80 m, the friction coefficient gradually increases and approaches 0.58–0.60 near the end of the test. The lower initial friction compared with 0HEA indicates that the addition of 10 wt.% HEA restricts, to some extent, extensive plastic deformation of the Cu-rich matrix. Previous studies on HEA-reinforced Cu matrices have similarly shown that hard HEA particles can increase matrix hardness and provide load-bearing support, thereby improving resistance to wear and plastic deformation [46,49]. The relatively large-amplitude peaks in the friction curve are consistent with intermittent contact instabilities associated with local cracking, disruption of smeared surface layers, temporary entrainment of detached wear debris into the contact, and interaction of the counterface with exposed hard particles. Hard reinforcement particles exposed at or detached from a Cu-based matrix have been reported to modify the instantaneous contact conditions and promote abrasive interactions [48].

A distinctly different friction response is observed for 20HEA. After a short running-in period, the friction coefficient rapidly reaches approximately 0.30–0.33 and subsequently remains mainly within the narrow range of approximately 0.30–0.38. In addition to exhibiting the lowest average coefficient of friction, 20HEA shows substantially smaller fluctuation amplitudes than the other compositions. These low-amplitude oscillations indicate a more stable sliding contact in which abrupt changes associated with adhesion, particle entrainment, delamination, and grooving are suppressed. This interpretation is consistent with previous studies showing that HEA reinforcement can improve the tribological stability of Cu matrices through increased hardness and load-bearing capability. In Cu–HEA systems specifically, the distribution and spacing of hard reinforcement particles have also been identified as factors governing surface contact and wear resistance [47]. For 30HEA, the friction coefficient is initially relatively high, reaching approximately 0.55–0.60 during the early stage of sliding, and then decreases markedly to approximately 0.38–0.40 at around 50–60 m. The high initial friction is consistent with a heterogeneous near-surface contact condition arising from the high HEA fraction, localized HEA clustering, residual porosity, and reduced continuity of the Cu matrix. During the early stage of sliding, these heterogeneities promote non-uniform asperity contact, localized deformation, and strong interaction between the steel counterface and exposed hard regions. As sliding progresses, the surface asperities are gradually flattened, plastic accommodation and possible strain hardening develop in the surrounding Cu-rich regions, while loose wear debris is redistributed or compacted within local surface depressions. These processes promote a more conformal contact geometry and account for the pronounced decrease in friction observed after approximately 50–60 m.

**Figure 15:**
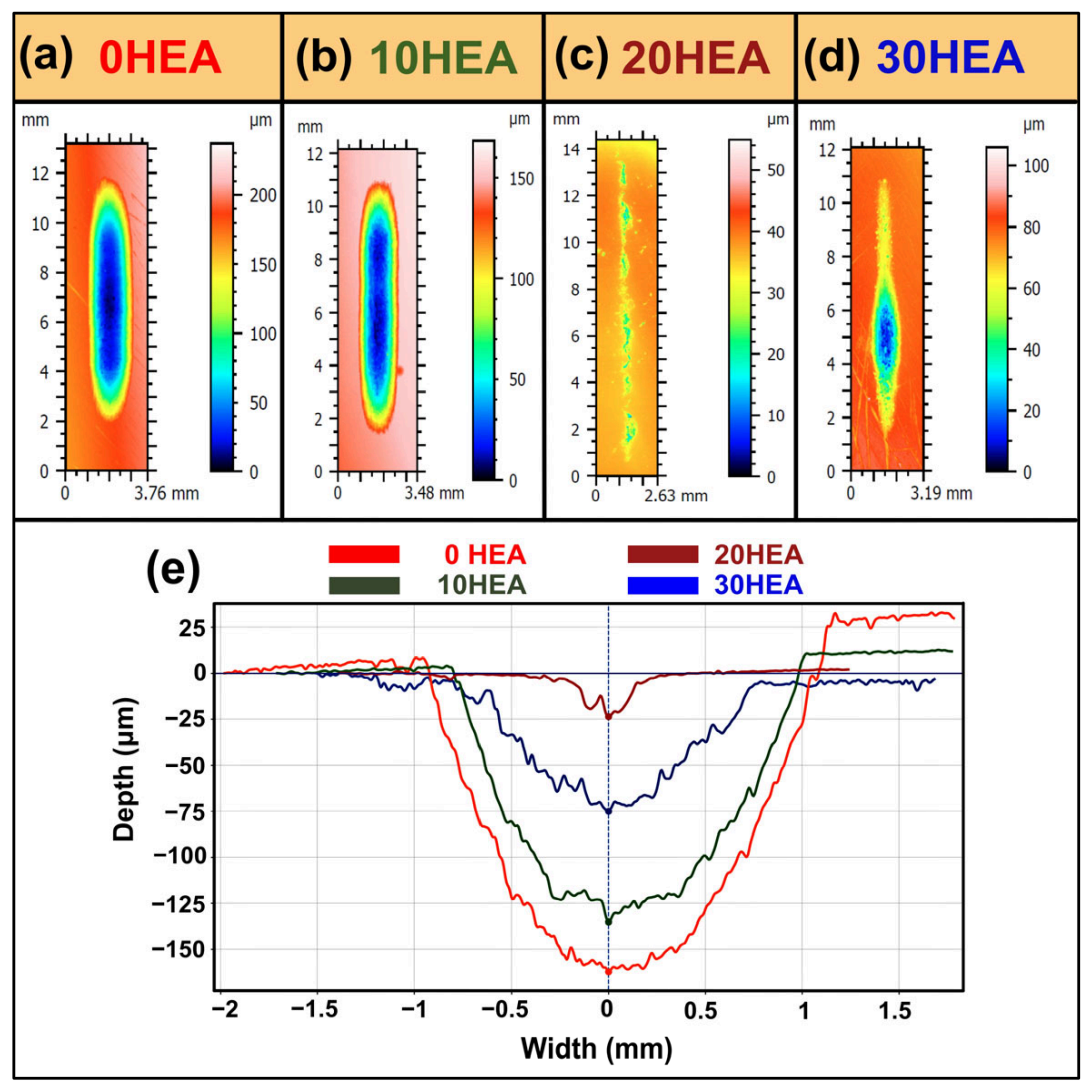
2D wear surface topographies of (**a**) 0HEA, (**b**) 10HEA, (**c**) 20HEA, and (**d**) 30HEA, and (**e**) representative transverse wear-depth profiles.

The 2d wear-track topographies and transverse profiles presented in Figure 15 closely correspond to the observed friction behavior. The 0HEA sample exhibits the widest and deepest wear scar, with an apparent track width of approximately 3.76 mm and a maximum depth approaching 160 µm (Figure 15e). Considerable material accumulation above the original surface level is also observed along the track edges in the transverse profile, indicating extensive plastic displacement and pile-up of the Cu-rich matrix (Figure 15a). Such behavior is consistent with the relatively low hardness of Cu, which facilitates plastic deformation and material displacement during sliding. For the 10HEA composite, the wear-track width decreases to approximately 3.48 mm, while the maximum depth is reduced to approximately 130–140 µm (Figure 15a–e). Although these values indicate an improvement compared with 0HEA, substantial penetration and plastic displacement of the matrix are still evident. The most pronounced reduction is observed for 20HEA, where the wear-track width is limited to approximately 2.63 mm and the maximum depth to only about 20–25 µm (Figure 15c). The corresponding profile is markedly shallower and less extensively deformed than those of the other compositions, indicating that the reduced friction coefficient is accompanied by a substantial decrease in surface penetration and material removal. When the HEA content is increased to 30 wt.%, the wear-track width increases to approximately 3.19 mm and the maximum wear depth reaches approximately 70–75 µm. Although these values remain substantially lower than those of 0HEA and 10HEA, they are considerably higher than those measured for 20HEA.

**Figure 16:**
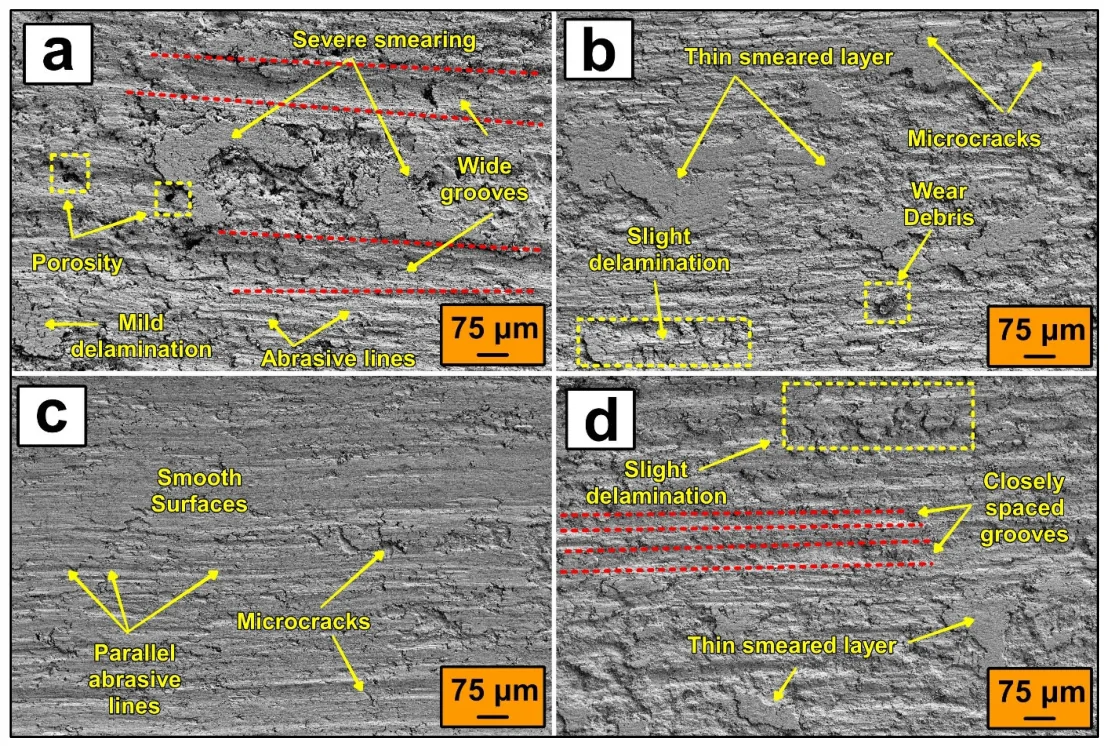
SEM images of the worn surfaces of (**a**) 0HEA, (**b**) 10HEA, (**c**) 20HEA, and (**d**) 30HEA composites after reciprocating dry sliding.

**Figure 17:**
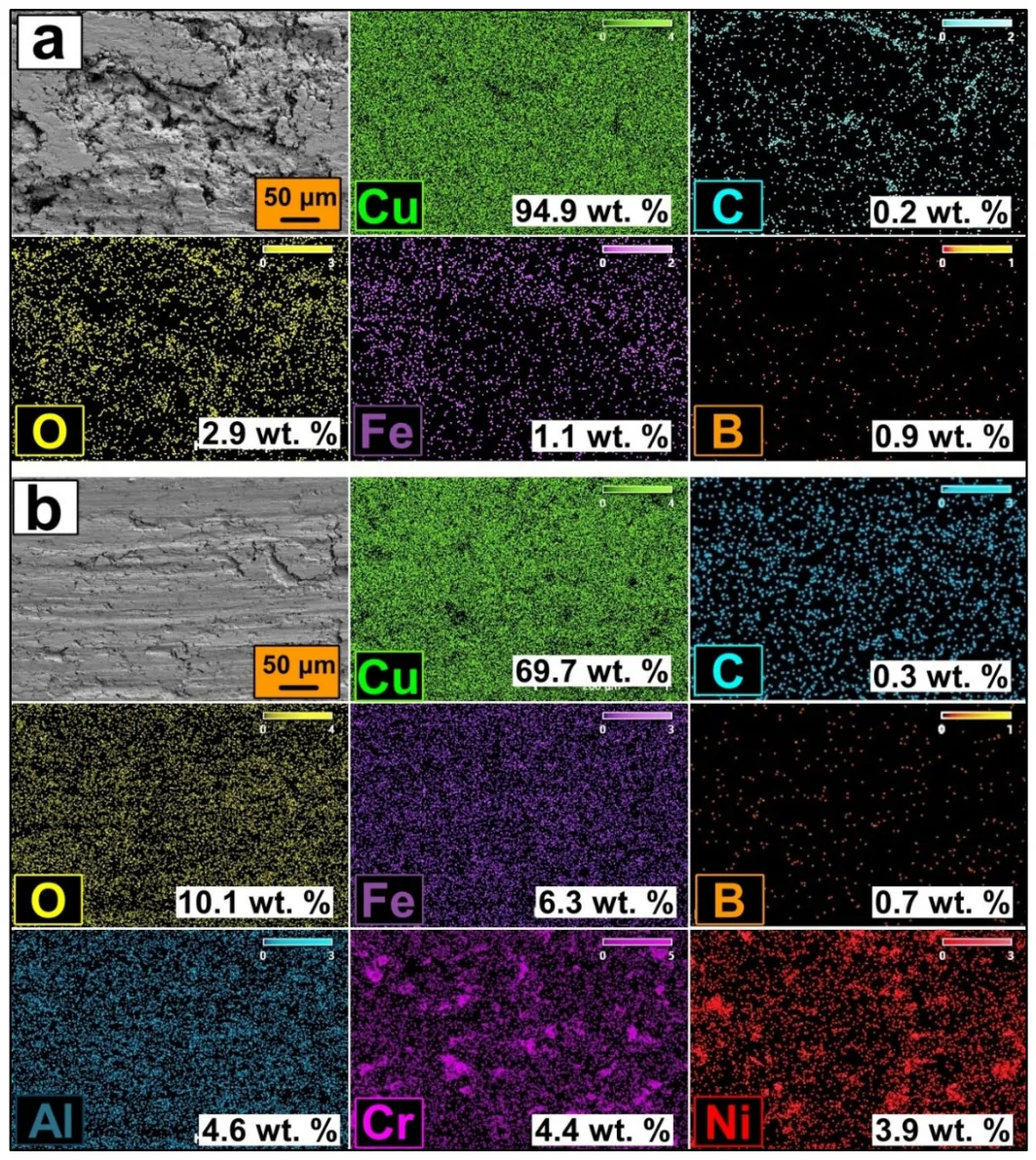
SEM/EDS elemental maps of representative worn regions of (**a**) 0HEA and (**b**) 20HEA composites.

SEM images of the worn surfaces obtained after the wear tests and EDS elemental mapping analyses of the worn surfaces of the 0HEA and 20HEA samples are presented in Figure 16 and Figure 17, respectively. As shown in Figure 16a, the worn surface of the 0HEA sample is characterized by severe smearing, broad grooves parallel to the sliding direction, abrasive scratches, and localized delamination. The coexistence of smearing and grooving indicates a mixed wear mechanism involving adhesive deformation of the Cu-rich surface together with abrasive grooving caused by wear debris entrapped within the contact. Repeated deformation of the smeared layers promotes the development of subsurface cracking and localized delamination over time, thereby contributing to the relatively large wear depth measured by profilometry. According to the EDS maps in Figure 17a, the worn 0HEA surface consists predominantly of Cu (94.9 wt.%), accompanied by 2.9 wt.% O and 1.1 wt.% Fe. Since Fe is not a constituent of the 0HEA composite, its detection indicates material transfer from the 100Cr6 steel counterface to the composite surface during sliding. The simultaneous presence of Fe and O is consistent with the occurrence of tribo-oxidation within the contact region. The oxygen-rich mechanically mixed layer observed after sliding is more appropriately interpreted as a tribologically generated mixture containing material derived from the Cu–B_4_C–HEA composite, transferred material from the 100Cr6 counterbody, and oxygen incorporated during sliding. As shown in Figure 16b, the smeared layer on the 10HEA surface is markedly thinner, while the broad and severe grooves observed for 0HEA are less pronounced. Nevertheless, microcracks, localized wear debris, and slight delamination are still present. These observations indicate that HEA addition suppresses extensive plastic flow but does not completely eliminate cyclic surface damage. The presence of localized microcracks and detached material is also consistent with the relatively sharp fluctuations observed in the friction curve.

The worn surface of 20HEA is considerably smoother (Figure 16c). Only fine, predominantly parallel abrasive scratches and isolated microcracks are observed, whereas severe smearing, broad grooving, and extensive delamination are largely absent. This morphology indicates a transition from severe mixed adhesive–abrasive wear to a milder sliding regime dominated primarily by shallow abrasive wear. The hard and well-dispersed HEA particles restrict plastic displacement of the Cu matrix and reduce the penetration depth of the counterface. At the same time, the continuous matrix provides sufficient support to limit excessive detachment of the reinforcement particles. This combination is consistent with the shallow wear profile in Figure 15 and the highly stable friction response in Figure 14. For the 20HEA sample, the oxygen content within the worn region increases from 2.9 to 10.1 wt.%, indicating pronounced oxygen enrichment of the mechanically mixed layer formed during sliding (Figure 17b). Al, Cr, Ni, and Fe originating from the HEA phase are also distributed throughout the analyzed region. Although the simultaneous presence of these metallic elements and oxygen does not by itself confirm the formation of a discrete oxide film or permit identification of specific oxide phases, oxygen- and oxide-rich mechanically mixed layers have been reported in similar HEA-containing tribological systems [47,50,51]. Depending on their composition and stability, such layers may reduce direct metallic contact and material removal during sliding. However, because EDS cannot resolve chemical states or distinguish individual oxide phases, this mechanism cannot be directly assigned to the present system. The current results therefore support only the presence of an oxygen-rich mechanically mixed layer, whose occurrence coincides with the lower friction and wear of the 20HEA composite. The improved tribological response is primarily attributed to the mechanical stabilization provided by the hard and well-dispersed HEA reinforcement, while a possible contribution from the oxygen-rich mechanically mixed layer cannot be excluded. This coupled mechanism is consistent with the smoother worn-surface morphology in Figure 16c, the shallow wear scar in Figure 15c, and the low-amplitude friction fluctuations observed in Figure 14. The worn surface of the 30HEA sample contains closely spaced parallel grooves, a thin smeared layer, and regions of slight delamination (Figure 16d). The more pronounced parallel grooves compared with 20HEA indicate that abrasive grooving becomes more prominent in 30HEA. In contrast, the persistence of the smeared layer and delaminated regions shows that plastic deformation of the Cu matrix and adhesive interactions are not completely suppressed. Accordingly, the wear behavior of 30HEA is more appropriately described as a mixed wear regime in which abrasive wear becomes more pronounced while adhesive deformation and localized delamination remain active. The deterioration at 30 wt.% HEA demonstrates that increasing the fraction of the hard nanocrystalline reinforcement does not produce a monotonic improvement in tribological performance. Beyond an intermediate reinforcement level, the beneficial mechanical contribution of the HEA is increasingly offset by reinforcement clustering, residual porosity, and loss of Cu-matrix continuity.

**Figure 18:**
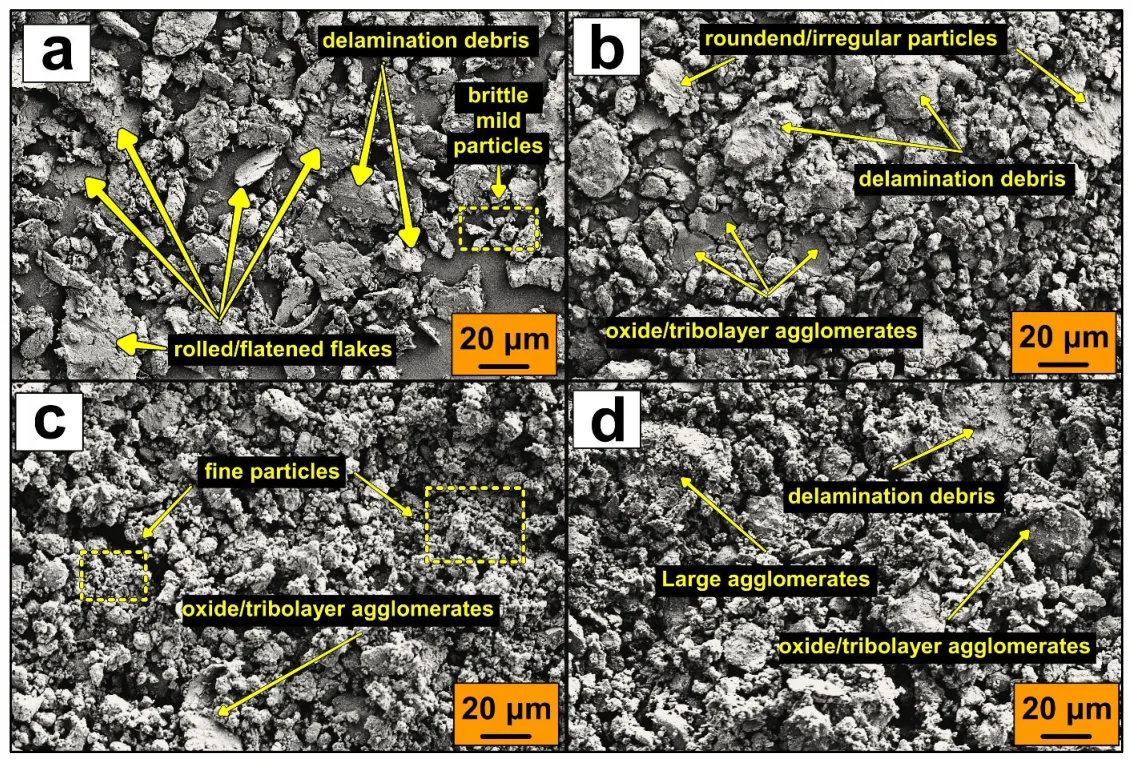
SEM images of wear debris collected from the (**a**) 0HEA, (**b**) 10HEA, (**c**) 20HEA, and (**d**) 30HEA samples.

The wear-debris morphologies in Figure 18 further support the wear mechanisms identified from the worn surfaces. The 0HEA sample (Figure 18a) contains large rolled/flattened flakes and delamination fragments, reflecting extensive plastic deformation and adhesive–delamination wear of the Cu-rich matrix. With 10 wt.% HEA (Figure 18b), the debris becomes smaller and more irregular, although delamination fragments and agglomerated tribolayer debris remain, indicating that severe plastic flow is reduced but not fully suppressed. The 20HEA sample (Figure 18c) exhibits predominantly fine particles and compact oxide/tribolayer-type agglomerates, consistent with the smoother wear surface, shallow abrasive scratches, and oxygen-rich mechanically mixed layer observed for this composition. At 30 wt.% HEA (Figure 18d), larger agglomerates and renewed delamination debris appear, consistent with the re-emergence of mixed abrasive–adhesive wear associated with HEA clustering, residual porosity, and reduced matrix continuity.

**Figure 19:**
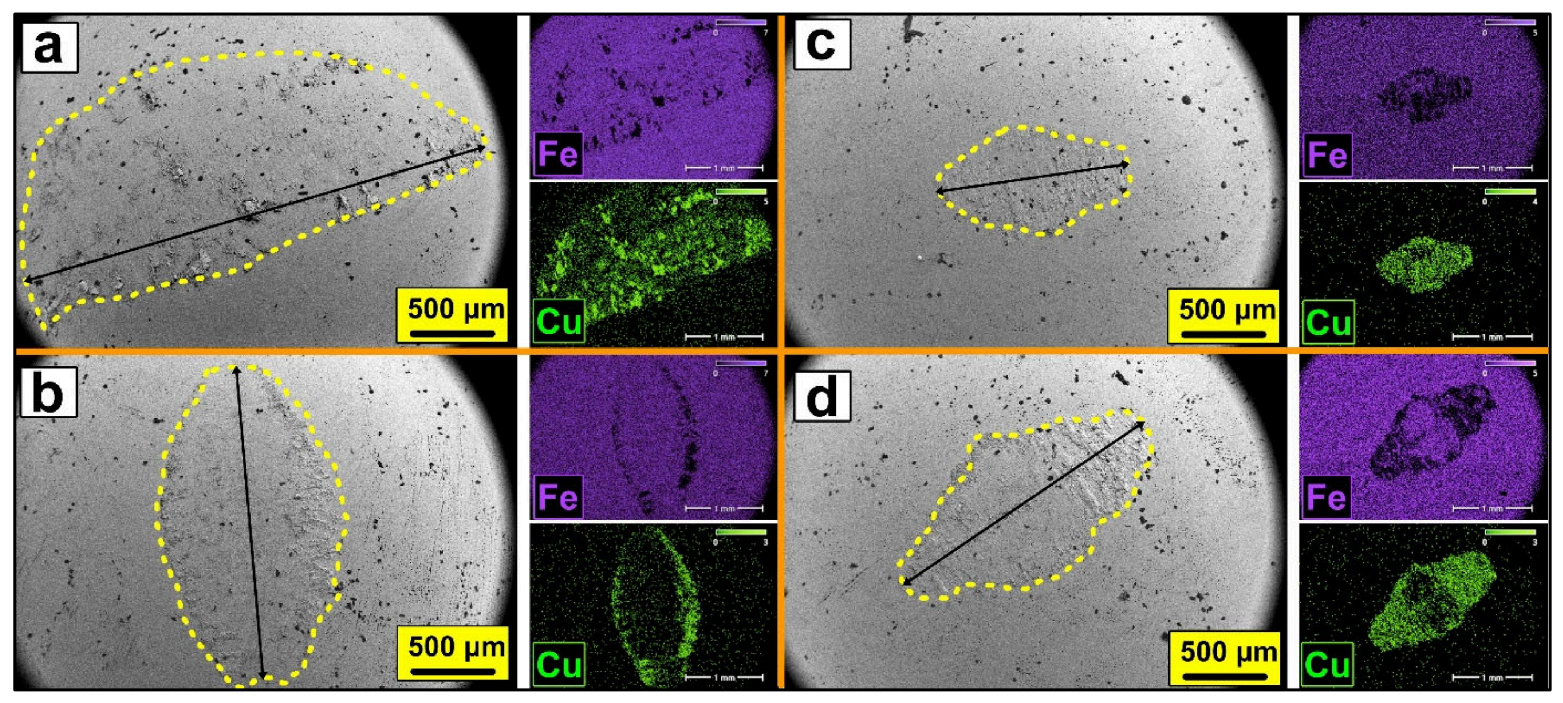
SEM images and corresponding Fe/Cu EDS maps of the 100Cr6 counterface wear scars after sliding against (**a**) 0HEA, (**b**) 10HEA, (**c**) 20HEA, and (**d**) 30HEA samples.

Figure 19 presents the SEM images of the wear scars formed on the 100Cr6 steel counterface together with the corresponding Fe and Cu EDS maps after sliding against the 0HEA, 10HEA, 20HEA, and 30HEA composites. The counterface SEM/EDS analysis and the wear-scar dimensions reported in Table 2 were obtained from one representative counterface corresponding to each composition. Accordingly, these dimensions are used as complementary geometrical indicators of counterface damage rather than as statistically averaged wear parameters; the statistical reproducibility of the tribological response is instead represented by the three repeated wear tests and the corresponding variability in specific wear rate. The Cu maps reveal distinct Cu-rich regions within the contact scars, confirming transfer of material from the Cu-based composites to the steel counterface during sliding. The size of the affected region changes markedly with HEA content, with the largest scar observed for 0HEA and the smallest for 20HEA. The quantitative measurements in Table 2 support these observations. The counterface wear-track width decreases from 2065 µm for 0HEA to 1718 µm for 10HEA and reaches a minimum of 742 µm for 20HEA. Similarly, the contact area decreases from 2.778 to 1.575 and 0.481 mm^2^, respectively. At 30 wt.% HEA, both parameters increase again to 1086 µm and 1.061 mm^2^. A clear correspondence is nevertheless observed between the representative counterface damage and the independently measured composite wear response. From 0HEA to 20HEA, the specific wear rate decreases by approximately 88%, while the counterface wear-track width and contact area decrease by approximately 64% and 83%, respectively. Conversely, from 20HEA to 30HEA, the specific wear rate increases by approximately 119%, accompanied by increases of approximately 46% in counterface wear-track width and 121% in contact area. Although these counterface measurements are representative rather than statistically averaged values, the consistent evolution of all three parameters supports the proposed relationship between reduced composite material removal and decreased counterface damage. This trend is also consistent with the corresponding wear-track profiles of the composites. The 0HEA and 10HEA samples exhibit relatively wide and deep wear tracks, whereas 20HEA shows the narrowest track and the lowest maximum wear depth of approximately 20–25 µm. In contrast, increasing the HEA content to 30 wt.% increases the wear-track width and depth again. Thus, the minimum counterface contact area and Cu-transfer region observed for 20HEA are consistent with its shallow wear scar and reduced material removal, indicating that 20 wt.% HEA provides the most favorable tribological response among the four compositions investigated in the present study. This should not be interpreted as establishing 20 wt.% as an absolute optimum HEA content, since additional intermediate compositions would be required to define the precise optimum reinforcement fraction.

## 4. Conclusions

Cu–1 wt.% B_4_C composites containing 0, 10, 20, and 30 wt.% mechanically alloyed AlCrFeCuNi HEA reinforcement were fabricated by spark plasma sintering. The main conclusions are as follows:

(1) Increasing the HEA content systematically modified the powder characteristics, densification, hardness, electrical conductivity, and tribological response of the Cu–1 wt.% B_4_C composites. The beneficial effect of increasing the HEA content was not monotonic because the higher reinforcement fractions progressively reduced Cu-matrix continuity and densification.
(2) Among the four HEA levels investigated, the 20HEA composite exhibited the best overall property balance, reaching a hardness of 138.94 HB, a relative density of 96.1%, and an electrical conductivity of 67% IACS, while also providing the lowest friction coefficient and specific wear rate. The present results therefore identify 20 wt.% as the best-performing composition within the investigated range, rather than as an absolute optimum HEA fraction.
(3) The same composition exhibited the lowest specific wear rate of 1.01 × 10^−3^ mm^3^/N·m and an average friction coefficient of 0.36. The wear rate was approximately 88% lower than that of 0HEA, and the maximum wear-track depth was limited to approximately 20–25 µm.
(4) The improved tribological response of 20HEA was associated with the combined effects of the hard mechanically alloyed HEA reinforcement, preservation of a continuous Cu-rich matrix, effective load support, and presence of an oxygen-rich mechanically mixed surface region observed after sliding
(5) Increasing the HEA content to 30 wt.% promoted reinforcement clustering, HEA–HEA contacts, residual porosity, and disruption of Cu-matrix continuity. These microstructural changes partially offset the strengthening effect of the additional HEA and increased both friction and wear relative to 20HEA.
(6) The results demonstrate that the performance of Cu–B_4_C composites reinforced with AlCrFeCuNi HEA is governed by a balance between reinforcement-induced strengthening and preservation of matrix continuity rather than by reinforcement content alone.

The present findings should be considered within the experimental scope of this study. Because only four HEA contents (0, 10, 20, and 30 wt.%) were investigated, the 20HEA composition represents the best overall property balance among the compositions tested rather than a precisely established optimum reinforcement fraction. In addition, the tribological ranking and wear-mechanism interpretations are restricted to the fixed ASTM G133 reciprocating dry-sliding condition employed here and may change under different loads, sliding conditions, or environments.

## Figures and Tables

**Table 1:** The sample codes and milling parameters.

Sample Code	Milling Time (h)	Reinforcement Content			Milling Speed (rpm)	Ball-to-Powder Weight Ratio
		HEA (wt.%)	HEA (vol.%)	B_4_C (wt.%)		
0HEA	2	0	0	1	400	10:1
10HEA	2	10	14.28	1	400	10:1
20HEA	2	20	27.19	1	400	10:1
30HEA	2	30	38.93	1	400	10:1

**Table 2:** Contact area and wear-track width measured on the 100Cr6 counterface ball after the reciprocating sliding tests.

Sample Code	Wear-Track Width (µm)	Contact Area (mm^2^)
0HEA	2065	2.778
10HEA	1718	1.575
20HEA	742	0.481
30HEA	1086	1.061

## Data Availability

The original contributions presented in the study are included in the article. Further inquiries can be directed to the corresponding author.
